# Marine-Derived Bioactive Metabolites as a Potential Therapeutic Intervention in Managing Viral Diseases: Insights from the SARS-CoV-2 In Silico and Pre-Clinical Studies

**DOI:** 10.3390/ph17030328

**Published:** 2024-03-01

**Authors:** Queency N. Okechukwu, Feyisayo O. Adepoju, Osman N. Kanwugu, Parise Adadi, Ángel Serrano-Aroca, Vladimir N. Uversky, Charles Odilichukwu R. Okpala

**Affiliations:** 1 Institute of Chemical Technology, Ural Federal University Named after the First President of Russia B. N. Yeltsin, Mira Street 28, Yekaterinburg 620002, Russia; 2 ARC Centre of Excellence in Synthetic Biology, School of Natural Sciences, Macquarie University, Sydney, NSW 2109, Australia; 3 Department of Food Science, University of Otago, Dunedin 9054, New Zealand; 4 Biomaterials and Bioengineering Lab, Centro de Investigación Traslacional San Alberto Magno, Universidad Católica de Valencia San Vicente Mártir, 46001 Valencia, Spain; 5 Department of Molecular Medicine, Morsani College of Medicine, University of South Florida, Tampa, FL 33612, USA; 6 UGA Cooperative Extension, College of Agricultural and Environmental Sciences, University of Georgia, Athens, GA 30602, USA

**Keywords:** SARS-CoV-2, therapeutics, pandemic, marine metabolites, marine organisms, viruses, viral infection

## Abstract

Worldwide urbanization and subsequent migration have accelerated the emergence and spread of diverse novel human diseases. Among them, diseases caused by viruses could result in epidemics, typified by the severe acute respiratory syndrome coronavirus 2 (SARS-CoV-2) which hit the globe towards the end of December 2019. The global battle against SARS-CoV-2 has reignited interest in finding alternative treatments for viral infections. The marine world offers a large repository of diverse and unique bioactive compounds. Over the years, many antiviral compounds from marine organisms have been isolated and tested in vitro and in vivo. However, given the increasing need for alternative treatment, in silico analysis appears to provide a time- and cost-effective approach to identifying the potential antiviral compounds from the vast pool of natural metabolites isolated from marine organisms. In this perspective review, we discuss marine-derived bioactive metabolites as potential therapeutics for all known disease-causing viruses including the SARS-CoV-2. We demonstrate the efficacy of marine-derived bioactive metabolites in the context of various antiviral activities and their in silico, in vitro, and in vivo capacities.

## 1. Introduction

Anthropogenic activities, including intensive agriculture and globalization, among others, have eroded biodiversity worldwide [1,2,3], accelerating the emergence and spread of numerous new human diseases [4,5]. Emerging infectious diseases, especially those caused by viruses, pose a threat to global health capable of causing widespread mortality in pandemics or localized outbreaks with high fatality rates [6,7]. Over the past five decades, there has been a continuous discovery of new emerging viruses of zoonotic origin. The first such case was the Ebola virus, which initially occurred in 1976 in Zaire and Sudan, and since then, there has been an ongoing report of Ebola outbreaks [8]. In 1981, the first case of what would become known as AIDS was recorded as *Pneumocystis carinii* pneumonia, primarily among homosexual males in the United States, heralding the onset of the AIDS epidemic; the causative agent, a retrovirus, was subsequently identified in 1983 [9]. The turn of the millennium witnessed the emergence of novel coronaviruses, with the Severe Acute Respiratory Syndrome (SARS) outbreak originating in Hong Kong in 2003. This was followed by the Middle East Respiratory Syndrome (MERS) in Saudi Arabia in 2012 [10]. In December 2019, the city of Wuhan in China’s Hubei Province became the epicenter for an outbreak of a pneumonia-like illness of unknown etiology, which was later identified as COVID-19, caused by a novel coronavirus designated SARS-CoV-2 [11].

Beyond their profound mortality and socioeconomic impacts, infectious diseases caused by emerging viruses represent escalating threats to global health. Accordingly, developing robust antiviral treatments and pre-emptive measures against potential pandemics has become a global public health priority [5,12]. Currently, vaccines and antiviral drugs are the primary interventions employed for the prevention and treatment of human viral infections. Vaccines are regarded as the most effective method for preventing viral infections [13]. Despite intensive research on a variety of viral pathogens, including the recent coronavirus strains, the repertoire of available antiviral treatments remains limited, compounded by the concerning decline in efficacy over time against certain viruses [14,15,16].

Since the approval of idoxuridine, the first antiviral drug, numerous others have been developed. However, the high mutation rate and genetic diversity of viruses often leads to treatment failure and rapid development of drug resistance [17]. Another significant concern is the cytotoxicity associated with these antiviral agents, which can limit their therapeutic utility [17]. On the other hand, vaccination is heralded as the most potent preventive strategy against viral infections, yet its effectiveness is not uniform across all populations, particularly among older adults, necessitating supplemental antiviral therapies [18]. This was illustrated by a community-wide serosurvey assessing the effectiveness of the BNT162b2 and CoronaVac vaccines against the SARS-CoV-2 Omicron variant over 100 days. The results of the study showed that at 100 days, vaccine effectiveness decreased to 26 and 35% for 3 and 4 doses of BNT162b2 and 6 and 11% for 3 and 4 doses of CoronaVac vaccines [15]. Given these challenges, it is crucial to explore and develop new therapeutic agents, particularly from natural sources such as marine-derived metabolites.

Over the years, natural products have provided resources/ingredients for developing drugs to treat and manage many human diseases. Covering over 70% of the earth’s surface, oceans are home to a wide array of organisms, thus providing a unique source of various metabolites with significant health benefits [19]. Research on marine microorganisms has steadily expanded since it started in the 1960s as a new area of study for natural products [20]. The unique secondary metabolites found in marine organisms with a variety of biological functions have evolved because of ecological stresses such as competition for space, surface fouling, predation, and successful reproduction [21]. For a long time, it was mainly disregarded how crucial these secondary metabolites are in the regulation of pathogenic and parasitic organisms. However, with improved extraction and characterization technologies, secondary metabolites can be sourced from marine organisms (both micro and macro).

Researchers have successfully isolated over 12,000 novel metabolites and continue to discover hundreds of new compounds annually from marine organisms, yielding new and potent natural bioactive ingredients [21,22]. On one hand, the terrestrial environment contains various plant-derived natural ingredients and molecules used as medicines; however, on the other hand, the marine ecosystem offers more untapped species of organisms from which potential bioactive natural compounds may be isolated [23]. These metabolites exhibit many biological activities of great pharmacological potentials, such as antimicrobial, antifungal, antifertility, antibiotic, and anticarcinogenic, and may serve as prophylaxis and treatment of human diseases.

Marine microorganisms, a subclass of marine organisms are recognized for their ability to produce antiviral agents, and they may offer limitless biological resources for obtaining therapeutic medications intended to treat and manage viral diseases in humans, as well as an endless supply of innovative compounds with promising medicinal properties and significant market potential [20]. Marine fungi alone yield between 150 and 200 novel molecules per year, including sesquiterpenoids, polyketides, and alkaloids [24]. Donia and Hamann reported the inhibitory potential of these marine-derived bioactive compounds against herpes simplex virus 1, poliovirus, yellow fever, dengue virus, rhinovirus, vesicular stomatitis virus, influenza viruses, and HIV-1 [21] with Griffithsin (a lectin extracted from red algae), suggested for anti-HIV activity, in clinical trials [19]. Furthermore, the structural engineering of these compounds by adding different functional groups (e.g., amines and ketones) and introducing double bonds, as well as the use of polymeric nanosystems, can bring about improved antiviral properties [25,26]. These agents, targeting various stages of the viral replication cycle, offer promising avenues for both therapeutic intervention and prophylactic measures against viral diseases, including COVID-19.

Thus, this perspective review discusses prospective marine-derived bioactive metabolites that may serve as therapeutic interventions in managing/treating various viral diseases. In addition, promising marine metabolites with potential inhibitory effects specifically against the replication mechanism of SARS-CoV-2 main proteases according to literature studies of molecular docking and simulation (in silico studies), coupled with in vitro and in vivo studies, are further discussed.

## 2. Metabolites from Marine Organisms

The marine ecosystem remains a repository of taxonomically diverse groups of unexplored micro- and macro-organisms, compared to its terrestrial counterpart. The complex marine habitats are exposed to extreme conditions and ecological pressures, including competition for space, pollution, and predation, which have powered the evolution of an assortment of potential secondary metabolites with different biological activities [21]. Naturally occurring secondary metabolites remain the main source of active ingredients for new therapeutic agents. In this regard, secondary metabolites from marine organisms have attracted immense attention over the years as potential raw materials for new broad-spectrum therapeutics due to the large ecological diversity of biological species contained in the marine environment [27].

**Figure 1:**
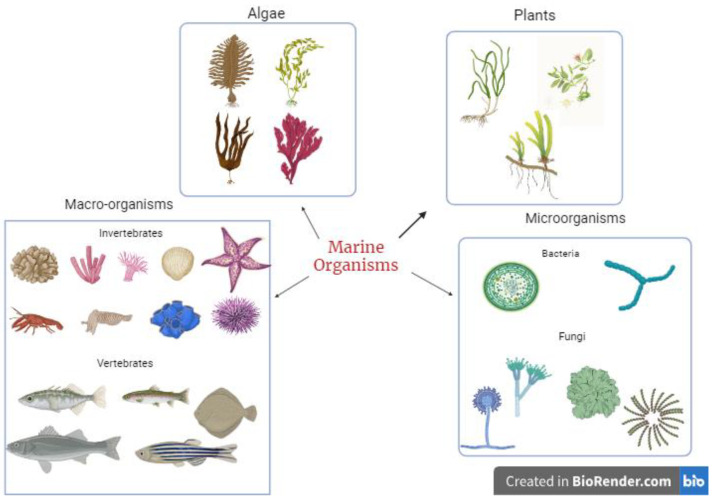
Sources of bioactive metabolites from marine organisms.

These secondary metabolites are synthesized by marine organisms as a survival and defence mechanism against other organisms, thus making them potential sources of bioactive compounds. With the emergence of various infectious diseases coupled with the menace of antibiotic resistance, marine organisms serve as a rich source of novel bioactive compounds for managing current and future viral diseases. The diversity of marine species (Figure 1) allows all kinds of potent metabolites to be isolated and tested for their potential pharmacological benefits to humans. Some bioactive compounds isolated and identified from marine organisms include terpenes, peptides and proteins, polysaccharides, lipids, alkaloids, and macrolides. The range of these compounds is related to the diverse mechanisms used by marine organisms to increase survival.

## 3. General Antiviral Activities of Metabolites from Different Marine Sources

### 3.1. Marine Microorganisms

#### 3.1.1. Bacteria

Marine microorganisms are microbes that exist in the marine, brackish water (coastal estuaries), or seawater (oceans, seas) habitats, and they include prokaryotes (i.e., bacteria and archaea) and eukaryotes (i.e., protists and fungi). There are several bacterial phyla in marine ecosystems, including the economically and biotechnologically important actinobacteria. However, the predominant novel compounds with potential biological activity are sourced from the genus *Streptomyces* [28,29]. Marine bacteria possess physiological and molecular characteristics that differ from their terrestrial counterparts, due to their symbiotic relationships with sponges, octocorallia, ascidians, and marine plants. Thus, they produce bioactive secondary metabolites for chemical defence by associated microflora (symbionts) or to survive in extreme environmental conditions [30,31]. The myriad of bioactive natural compounds extracted from marine bacteria has significantly increased in recent years. Moreover, marine bacteria continue to be a prolific source of bioactive compounds (i.e., peptides, exopolysaccharides, polyketides, and macrolatones) to treat/manage many disease conditions [29].

Table 1 shows some of the reported antiviral compounds of marine bacteria. A novel compound, antimycin A1a (**1**), extracted from *Streptomyces kaviengensis* was effective against western equine encephalitis virus (WEEV; half maximal inhibitory concentration, IC_50_ = 4 nM) [32]. The compound inhibited the cellular electron mitochondrial transport chain and suppressed de novo pyrimidine synthesis [32]. Others have reported that furan-2-yl acetate (**2**) isolated from *Streptomyces* VITSDK1 spp. inhibited fish noda virus, an important viral pathogen in cultured marine fishes [33]. Elsewhere, the heterogenous expression of type III polyketide synthase gene *vioA* (from deep-sea-derived *Streptomyces somaliensis* SCSIO ZH66) enhanced synthesis of antiviral methylated violapyrones (VLPs Q-T) (**3**–**6**) in *Streptomyces youssoufiensis* OUC6819. The resultant VLPs Q-T (**3**–**6**) showed inhibitory activity against strains of influenza A (IC_50_ = 30.6–68.4 μM against strain H1N1 and 45.3–95.0 μM with regards to strain H3N2) compared to ribavirin and non-methylated VLPs [34]. Butenolide analogue 3 (**7**) isolated from marine *Streptomyces* sp. AW28M48 showed anti-adenoviral activity at EC_50_ of 91 μM with no prominent cytotoxicity effects at 2 mM [35]. Furthermore, a marine exopolysaccharide (EPS) (**8**) extracted from *Pseudoalteromonas* spp. exerted a remarkable antiviral activity against herpes simplex (HSV-1) [36].

#### 3.1.2. Marine Fungi

Marine fungi are also a rich source of natural bioactive compounds with various biological activities, including antiviral effects (Table 2, Figure S1). It has been reported that marine fungi have developed specific metabolic pathways compared to their terrestrial counterparts due to salinity, high pressure, temperature change, and competition [41]. Several novel natural bioactive compounds of pharmacological relevance have been isolated, with prospects as the active ingredient(s) for new antiviral drugs [29]. The most studied marine fungi are of the genus *Penicillium*, which produces metabolites such as penicillin, griseofulvin, sorbicillacton A–B, isocoumarins, and sesquiterpenoids. These metabolites have shown a broad range of antiviral activities against important viruses, including the influenza virus (H1N1, H3N2), Newcastle disease virus (NDV), infectious brucellosis disease virus (IBDV), HIV, enterovirus 71 (EV-71), and herpes virus (HSV-1, HSV-2) [20,42,43]. A novel compound, rubrolide S (**13**), first isolated from marine-derived *Aspergillus terreus* OUCMDZ-1925, inhibited H1N1 virus at IC_50_ value of 87.1 μM with ribavirin as positive control (IC_50_ = 118.8 μM) [44]. Others have, likewise, reported that sorbicatechol A (**14**), extracted from the marine fungus *Penicillium chrysogenum* PJX-17, exerted an inhibitory effect against the H1N1 virus with IC_50_ values of 85 μM, respectively [45]. Similarly, pulvic acid (**15**) (94.4 μM) from *Aspergillus terreus* Gwq-48 inhibited the growth of the H1N1 virus [46]. Others reported new novel antiviral compounds (chrodrimanins K (**16**) and N (**17**)) and 3-hydroxypentacecilide A (**18**) secreted by *Penicillium* sp. SCS-KFD09. These compounds showed antiviral activity against H1N1 with IC_50_ values of 74, 58, and 34 μM compared to ribavirin as a positive control (IC_50_ = 103 μM) [47]. Similarly, polyketones (i.e., asteltoxin E (**19**) and F (**20**)) isolated from *Aspergillus* sp. SCSIO XWS02F40 inhibited H3N2 virus growth with IC_50_ values of 6.2 ± 0.1 and 8.9 ± 0.3 μM, respectively. In addition, asteltoxin E (**19**) inhibited the growth of the H1N1 virus (IC_50_ = 3.5 ± 1.3 μM) [48]. Besides these, five new indole diterpenoids (**21**–**25**), isolated from the fermented culture broth of *Penicillium camemberti* OUCMDZ-1492, exert inhibitory activity against H1N1 virus (IC_50_ values in the range of 28–40 μM) compared with ribavirin [49]. In another study, a novel tetrapeptide (asperterrestide A (**26**)), synthesized from marine *Aspergillus terreus* SCSGAF0162, inhibited the growth of H1N1 and H3N2 viruses with IC_50_ values of 15 and 8.1 μM, respectively [50].

#### 3.1.3. Marine Algae

Marine algae are photosynthetic plant-like organisms that are categorized into three groups: microalgae, macroalgae, and multicellular organisms. Microalgae are tiny unicellular microorganisms that form the phytoplankton and comprise approximately 50,000 species. Conversely, sea algae, also known as seaweed, have become prominent in the food and cosmetic industries due to their nutrients and rich sulfated polysaccharides content, among others, which have been shown to exert various biological activities such as anticancer, antioxidant, immunoregulatory, antiviral, antithrombic, and anti-inflammatory properties [77].

Microalgae are a repository of bioactive secondary metabolites; however, only a few studies were found to have reported the antiviral activity of marine microalgae-derived compounds (Figure S3). For instance, monogalactosyl diacylglyceride (**77**) extracted from *Coccomyxa* sp. showed a potential anti-HSV-2 activity [78]. Another study showed that exopolysaccharides (**78**) isolated from *Porphyridiumn cruentum* exerted viral-induced cytopathogenicity against HSV, VSV, and vaccinia virus [79]. A sulfated polysaccharide (p-KG03) (**79**) isolated from *Gyrodinium impudicum* also inhibited influenza virus A (EC_50_ = 0.2–0.5 μg/mL) [80]. Marennine (**80**), a blue pigment isolated from diatom *Haslea ostrearia*, also inhibited herpes virus (HSV-1) [81].

Furthermore, a lectin with 121 amino acids (griffithsin (**81**)), extracted from *Griffithsia* sp. (red alga), showed potential antiviral activity against HIV [19]. Others have demonstrated the antiviral activity of sulfated polysaccharides (**82**) extracted from *Grateloupe filicina*, *Ulva pertusa*, and *Sargassum qingdaoense* against AIV (H9N2 subtype) in in vivo and in vitro studies [82]. Similarly, sulfated glucuronorhamnan (**83**), extracted from *Monostoma nitidum*, showed a broad spectrum of antiviral activity against different viruses including enterovirus 71 [83]. Aguilar-Briseño et al. [84] also demonstrated that ulvan (**84**) and fucoidan (**85**) extracted from *Ulva clathrata* and *Cladosiphon okamuranus* inhibited Newcastle disease virus (NDV) in poultry. Another study showed that dieckol (**86**) a phlorotannin extracted from *Ecklonia cava* inhibited the activity of SARS CoV 3CLpro (IC_50_ = 2.7 μM) [85]. Additionally, extracts of *Ulva fasciata*, and *Codium decorticatum* exhibited 99.9% inhibitory activity against the HSV-1 virus. Similarly, the extract of *Laurencia dendroidea* exhibited 97.5% inhibitory activity against the HSV-1 virus [86]. Also, extracts of *Penicillius capitatus* and *Stypopodium zonale* exerted potential antiviral activity against herpes simplex virus type 2, with 96% and 95.8% inhibition, respectively [86].

### 3.2. Marine Plants

Seagrasses are flowering plants that evolved from terrestrial plants and adapted to the marine environment. They serve as habitats for several threatened species, thus supporting the resilience of the coastal environment and maintaining genetic variations, with potential bioactive resources. Nonetheless, only a few studies have successfully isolated bioactive compounds from seagrasses with potential antiviral activity (Figure S4). For instance, 1 mg/mL of asebotin (**87**), trans-caffeic acid (**88**), and quercetin-3-O-β-D-xylopyranoside (**89**) extracted from Egyptian seagrass (*Thalassodendtron ciliatum*) exerted 96.6 ± 0.9%, 70 ± 0.98%, and 53 ± 0.77% inhibitory activity, respectively, against HSV-1 [87]. Further investigation of the seagrass by the authors revealed the presence of thalassodendrone (6′-O-rhamnosyl-(1‴ → 6″)-glucopyranosyl asebogenin (**90**), IC_50_ = 3.3 μM) and asebotin (**87**) (IC_50_ = 4.4 μM) with potential virucidal activity against influenza virus A [88]. Also, an in vitro study demonstrated that thalassiolin D (**91**) isolated from *Thalassia hemprichii* inhibited the protease activity of HCV (IC_50_ = 16 μM) [89]. Besides seagrasses, several compounds have been isolated from mangroves, including alkaloids, steroids, and flavonoids (Figure S4) [19]. For instance, HIV-1 integrase inhibitors (i.e., integracins A (**92**) and B (**93**)) have been isolated from the mangrove plant (*Sonneratia hainanensis*) [90]. Furthermore, thaixylomolin I (**94**), also known as a khayanolide, extracted from *Xylocarpus moluccensis*, exhibited anti-H1N1 activity with IC_50_ values of 77.1 μM [91].

### 3.3. Marine Macro-Organisms

#### 3.3.1. Marine Invertebrates

Marine invertebrates comprise several phyla including the cnidarians, mollusca, bryozoan, ascidians, echinoderms, and porifera. We demonstrate (Figure S5) the antiviral properties of peptides [92,93,94,95], macrodiolide [96], nortopsentin alkaloids [97], alkaloids [98,99,100], steroids [101,102,103], polysaccharide [104], enzyme [105], and naphthopyrones isolated from marine invertebrates [106].

Two polybrominated diphenyl ethers (3,5-dibromo-2-(2,4-dibromophenoxy)-phenol (**95**) and 3,4,5-tribromo-2-(2,4-dibromophenoxy)-phenol) (**96**) extracted from *Dysidea granulosa* showed anti-HBV activity by inhibiting the core promoter essential for viral replication [107]. Similarly, metachromin A (**97**) extracted from marine sponge *Dactylospongia metachromia* inhibited the core promoter activity of HBV [108]. Others have also demonstrated that manoalide (**98**) isolated from *Luffariella variabilis* inhibited NS3 RNA helicase (IC_50_ = 15 μM) and ATPase (IC_50_ = 70 μM) activities [109]. Psammaplin A (**99**) extracted from *Psammaplysilla* sp., *Poecillastra* sp., and *Jaspis* sp. likewise inhibited NS3 RNA helicase and ATPase activities with IC_50_ values of 17 and 32 μM, respectively [110].

Yu et al. [111] investigated the antiviral activity of two aapatamine alkaloids: 3-(phenethylamino)demethyl(oxy)aaptamine (**100**), and 3-(isopentylamino)demethyl(oxy)aaptamine (8) (**101**), isolated from *Aaptos aaptos*, and found that at 10 μM the compounds exerted anti-HIV-1 activity with 88.0 and 72.3% inhibitory activity, respectively. Likewise, leucetta alkaloids, i.e., kealiinine B (**102**) and its derivatives, N-(6,7-Dimethoxy-4-(4-methoxyphenyl)-1-methyl-1H-naphtho-[2,3-d]imidazol-2-yl)-1H-imidazole-1-carboxamide (**103**) and 1-(6,7-Dimethoxy-4-(4-methoxyphenyl)-1-methyl-1H-naphtho-[2,3-d]imidazol-2-yl)-1-phenylethan-1-ol)) (**104**) extracted from *Leucetta chagosensis* inhibited the activity of tobacco mosaic virus (TMV) [112].

Mollusca is another group of marine invertebrates with a diverse repository of bioactive natural compounds against human pathogens. It is reported that 1, 10, and 100 μg/mL of structural subunits of hemocyanin (RvH1 (**105**) and RvH2 (**106**)) and their isoforms (RvH1-a (**107**) and RvH2-e **(108**)) extracted from *Rapana venosa* decreased replication of Epstein-Barr virus in B-phenotype lymphoblastoid cells [113]. Using Vero cells, the anti-HSV-1 activity (EC_50_ = 40–50 nM) of purified hemocyanin (**109**) extracted from *Haliotis rubra* was further demonstrated [114]. Similarly, cavortin **(110**), a major haemolymph protein extracted from *Crassostrea gigas*, showed significant anti-HSV-1 activity [115]. According to Novoa et al. [116], myticin C (**111**) peptide from mussel (*Mytilus galloprovincialis*) inhibited ostreid herpesvirus 1 (OsHV-1). In addition, nanovesicles of myticin C (**112**) improved the antiviral activity against HSV-1 and HSV-2 [116].

Soft corals are similarly reported to be a rich source of bioactive metabolites ranging from norditerpenes to sesquiterpenoids [29]. Norcembranoids (5-episinuleptolide (**113**) and sinuleptolide (**114**)) isolated from soft Indian coral (*Sinularia kavarattiensis*) exhibited anti-Chikungunya virus (CHIKV) activity [117]. Furthermore, briarane-type diterpenoids, briacavatolide C (**115**) and F (**116**) extracted from Taiwanese octocoral (*Briareum excavatum*) inhibited the activities of human cytomegalovirus (HCMV) [118,119]. Polyhydroxylated sterol (**117**) and ceramide derivatives (**118**) extracted from *Sinualaria candidula* also exerted anti-influenza A (H5N1) activity [120].

Crustaceans are a diverse group of invertebrates that belong to the phylum Anthropoda and include prawns, lobsters, shrimps, and crabs, among others. A peroxinectin homolog (Sp-PX) (**119**) extracted from *Scylla paramamosain* enhanced anti-white-spot-syndrome-virus (WSSV) immune responses in mud crabs [121]. Another study investigated the peptide scygonadin (**120**) extracted from *Scylla paramamosain* against WSSV. The results showed a dose-dependent downregulation of IE1 transcripts in Hpt cells at 25 and 50 μM [122]. Likewise, isoforms of anti-lipopolysaccharide factors (Sp-ALF1 (**121**) and 2 (**122**)) from *Scylla paramamosain* showed potential antiviral activity against WSSV [123]. Recently, in vitro and in vivo studies using a peptide (LvHcL48 (**123**)) derived from *Litopenaeus vannamei* similarly demonstrated anti-WSSV activity. The authors suggested that LvHcL48 (**123**) bound to the viral envelope protein VP28 in WSSV and inhibited replication [124]. A new crustin (SpCrus6 gene (**124**)) extracted from *Scylla paramamosain* decreased the virion load of WSSV in mud crabs [125].

Another group of marine invertebrates are ascidians, which include tunicates and sea squirts. There are approximately 3000 recognized species of tunicates worldwide, and it is speculated that tunicates are reservoirs of novel biologically active compounds [126]. Studies have extracted and reported several pharmacologically active compounds from tunicates. For example, tris-phenethyl urea (molleurea A) (**125**), mollamides E (**126**) and F (**127**) extracted from *Didemnum molle* demonstrated anti-HIV activity. Also, mollamides F (**127**) and molleurea A (**125**) decreased HIV-1 replication with IC_50_ values of 78 and 60 μM, respectively [127]. Another study with prunolide A (**128**) and cadiolide B (**129**) isolated from the ascidian *Synoicum*, showed virucidal properties against Japanese encephalitis virus (JEV) [128]. A potential anti-HIV compound (divamide A (**130**)) was likewise extracted from *Didemnum molle* [129].

#### 3.3.2. Marine Vertebrates

Over past decades, some studies have reported the antiviral activities of peptides derived from marine vertebrates, especially fish. Peptide analogue Pa-MAP (**131**), isolated from Winter flounder (*Pleuronectes americanus*), inhibited HSV-1 and HSV-2 viruses in Vero cells [130]. Furthermore, Pa-MAP (**131**) from *P. americanus* exerted 90% inhibitory activity against HSV-1 with a selectivity index of 5 [131]. In addition, phospholipase A2 (**132**) extracted from *Pterois volitans* (PV-PLA2) showed 97.8% inhibitory activity against simian retrovirus serotype-2 (SRV2) in A549 cells [132].

Antiviral activities of other reported compounds isolated from marine macro-organisms (vertebrates and invertebrates) are summarized in Table 3.

## 4. SARS-CoV-2 Virology and Mode of Entry

**Figure 2:**
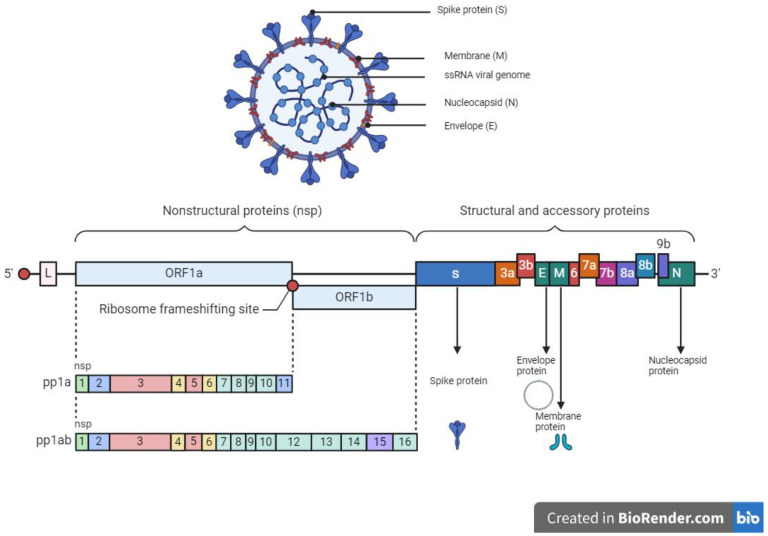
Structure and genome organization of SARS-CoV-2.

The SARS-CoV-2 which caused the severe acute respiratory coronavirus disease 2019 (COVID-19) belongs to the *Coronaviridae* family with a zoonotic potential, thus transmitted from humans and other mammals [134]. It shares a similar genomic sequence with the original SARS-CoV (~79.5% similarity) and BatCoV RaTG13 (~96% similarity). SARS-CoV-2 is a 50–200 nM positive-sense single-stranded enveloped RNA virus (+ssRNA) with a genome size of 28–30 kb [135,136,137]. The genome of SARS-CoV-2 consists of over 29,000 bases and codes for 29 proteins. Of the 29 proteins, the viral genome encodes 4 structural proteins and 16 non-structural replicate polyproteins which play a crucial role in the viral replication complex (Figure 2). The structural proteins include the spike (S) glycoprotein which binds to the host ACE2 receptor to initiate infection, small envelope (E) glycoprotein, and membrane (M) glycoprotein distributed along the viral envelope, and nucleocapsid (N) phosphoprotein which is an RNA-binding protein that facilitates the packaging of the genome and protects the viral genome [137,138]. Structural proteins are important for infection and replication in the host cell, thus making them ideal candidates or targets for antiviral therapies. The non-structural proteins (NsPs) are synthesized as long polypeptides which release the RNA-dependent RNA polymerase (RdRp), Nsp12, when activated by the main protease (MP), Nsp5. MP can be targeted by antiviral drugs against SARS-CoV-2, given its key role in virus replication and transcription [139,140].

Infection of host cells by SARS-CoV-2 transmission occurs via endocytosis, which involves the interaction of host cell surface receptors (fusion) with endosomal components. The spike proteins are essential for the entry of the virus into a host cell. Particularly, the S1/S2 subunit facilitates attachment to the cell and subsequent fusion. It requires initial priming by the transmembrane protease, serine 2 (TMPRSS2), cysteine protease, and cathepsin L (CatL). In the host cell, the angiotensin-converting enzyme 2 (ACE2), a type I membrane receptor protein found in the lungs and arteries [137], serves as the binding site for SARS-CoV. The SARS-CoV-2 virion attaches to the enzymatic domain of ACE2 on the surface of cells via the receptor-binding domain (RBD) of the S1 unit, and subsequently, the cell TMPRSS2 opens the S protein, allowing for the fusion of the S2 subunit and ACE2 [141]. This results in endocytosis and the translocation of both the virus and enzyme into endosomes [142]. The virus subsequently escapes when the pH of the endosome drops or is cleaved by cathepsin, thus releasing its RNA into the cell cytoplasm. The virus then replicates and spreads new copies of the virus to infect more cells [136,141].

### Therapeutic Target Site to Inhibit SARS-CoV-2 Entry and Replication

**Figure 3:**
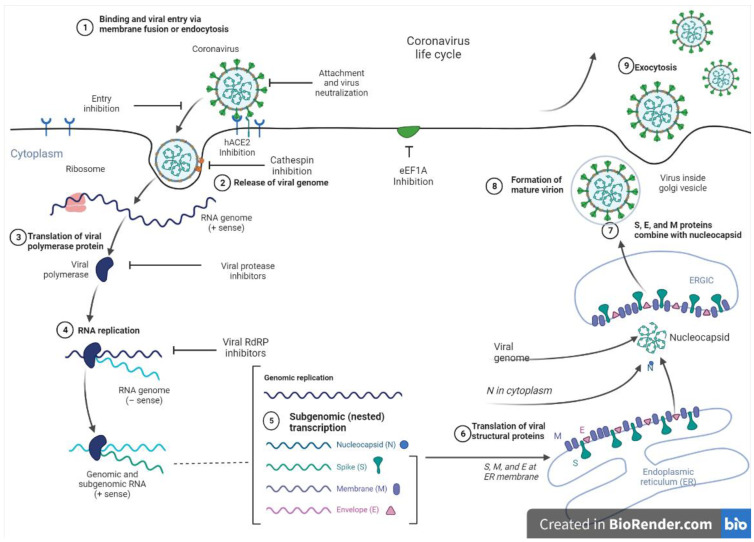
SARS-CoV-2 entry and replication cycle with potential inhibition sites for marine metabolites.

At present, vaccines, monoclonal antibodies, peptides, small molecule drugs, and interferon therapies serve as viable options to manage SARS-CoV-2. Nevertheless, targeting the replication machinery of the virus remains a promising therapeutic approach. In this regard, viral proteases are suitable targets, as these enzymes play critical roles in the replication of the virus by cleaving proproteins after translation into the host cell cytosol during viral protein maturation [143]. Figure 3 shows the entry and replication cycle of SARS-CoV-2 entry with inhibition sites of some marine-derived metabolites. SARS-CoV-2 spike (S) glycoprotein binds to the ACE2 receptor on the host cell surface, and the virus subsequently enters the cells via endocytosis to release its positive-sense ribonucleic acid (RNA) into the host cell. The viral genomic RNA is then transcribed and translated to produce non-structural proteins (nsps), including replicase polyproteins (RNA-dependent RNA polymerase and helicase), which then creates an RdRp complex. Within the RdRp complex, subgenomic transcription and RNA replication occur to synthesize negative-strand guide RNA (gRNA) and a set of subgenomic RNAs for viral replication and transcription. Subgenomic RNAs are synthesized and translated into viral structural proteins such as the spike (S), nucleocapsid (N), membrane (M), and envelope (E). After viral structural proteins are translated, S, E, and M proteins are processed in the Endoplasmic Reticulum-Golgi (ERG) intermediate compartment of the host cell. In the cytoplasm, nucleocapsids assemble and bud into the lumen of the ERG intermediate compartment. Finally, the mature virus inside the Golgi vesicle is exocytosed from the infected cell. Through ACE-2 receptors, a mature virus can infect the lung, endothelium, intestine, heart, testis, and kidney [140,144].

Overall, the ACE2 protein, transmembrane protease serine 2 (TMPRSS2), papain-like protease (PL2pro), and main protease (Mpro)/chymotrypsin-like protease (3CLpro), which are crucial for viral replication and proliferation in the human host, are all potential targets under investigation for therapeutic interventions against SARS-CoV-2 [134,135,137,143,145]. PL2pro and 3CLpro/Mpro cleave large polyproteins of SARS-CoV-2 before being proteolytically processed to generate the individual proteins required for viral replication [141,142]. 3CLpro/Mpro plays a leading role in transcription, releasing replicative proteins, including the viral RNA polymerase and helicase proteins [146,147]. 3CLpro/Mpro is the main protease found only in the coronavirus family and is considered the most suitable target for virus inhibition as it cleaves the coronavirus polyprotein at eleven conserved sites. It is worth noting that glycan-protein interactions are important during viral binding to the host cell, considering that glycosylation of the S-protein shields the proteins from immune recognition. Hence, disrupting S-protein glycosylation significantly impairs viral entry, thus serving as another potential target for vaccine development and therapeutic interventions [137]. Furthermore, neuropilin 1 (NRP1), a host protein, aids virus entry, making it an attractive target [148]. In addition, RNA-dependent RNA polymerase (RdRp), which catalyzes the replication of the viral RNA genome, is a probable target as well [149]. Also, more focus has been placed on decreasing the levels of ACE2, a part of the renin–angiotensin system that regulates blood pressure, given that it is the main entry point of the virus in humans. However, this may not be a good approach since it can alter the central pressure control system and cause stroke or other medical conditions [148].

## 5. Potential In Silico and Pre-Clinical Studies of Marine-Derived Metabolites against Target Sites of SARS-CoV-2 as Therapeutics

Various in vitro and in vivo anti-SARS-CoV and anti-MERS-CoV studies have been carried out using a wide myriad of bioactive compounds during the previous outbreaks. Considering that they share some similarities with SARS-CoV-2, some of these bioactive compounds may be repurposed to screen for their potential against SARS-CoV-2. Designing an efficient broad-spectrum antiviral therapy against coronaviruses is an efficient way to counter various mutant strains of SARS-CoV-2, which hinder the effectiveness of the current vaccines [150]. Natural bioactive compounds can be used to design new antiviral drugs against viral infections, coupled with boosting the innate immune system. However, there are challenges involved considering the diversity of natural metabolites, chemical intricacies, and different extraction methodologies [135].

To save time in screening bioactive compounds for potential activity, a virtual or computational screening approach is recommended. In this regard, in silico techniques such as molecular docking, molecular dynamics simulations, and network pharmacology are useful for the preliminary identification of natural compounds that can directly inhibit target proteins [5,135,151]. Molecular docking evaluates the binding and interaction between the specified molecules (i.e., marine-derived metabolites) and the target protein(s), whereas network pharmacology employs computationally simulated drug-targeted interactions to identify potential inhibitors for a particular target and mode of action [152,153]. Additionally, the process evaluates the stability of the predicted protein–ligand complex considering factors such as the nature of the solvent [154]. By including biological circumstances, such as structural motions and the 3D structure of the targets, more reliable affinity values of the metabolites are estimated [155]. Nevertheless, most studies about network pharmacology for SARS-CoV-2 are related to existing traditional drugs with limited studies available for marine-derived drugs [156,157].

### 5.1. In Silico, In Vitro, and In Vivo Studies of Major Classes of Metabolites against Entry and Replication of SARS-CoV-2

Diverse and unique polysaccharides, proteins, lipids, terpenoids, flavonoids, steroids, and alkaloids with virucidal activities have been extracted from marine organisms [158]. Some of these metabolites and their derivatives are reported to be protease inhibitors that can inhibit DNA and RNA viruses, and thus may serve as potential protease inhibitors against SARS-CoV-2 [5].

#### 5.1.1. Polysaccharides

Marine-derived polysaccharides are considered important biological macromolecules with unique and diverse structures and are considered valuable resources for drug discovery and design [159]. Moreover, marine-derived polysaccharides are cheaply available in nature, non-toxic, safe, biocompatible, and biodegradable [160].

a. Sulfated Polysaccharide (SP)

Found in the cell walls of marine microbes, sulfated polysaccharides (SP) are naturally occurring water-soluble complex polymers extracted using water as a solvent [159]. Others have speculated that SP-derived therapy may be used to manage COVID-19 disease because it prevents/inhibits adherence of the S-protein to the heparin sulfate co-receptor and thus decreases viral infection by acting as a decoy in host tissues [159,160]. Various concentrations of fucoidan (RPI-27 (**151**) and RPI-28 (**152**)) extracted from *Saccharina japonica* showed antiviral activity against SARS-CoV-2 in Vero cells. RPI-27 (**151**) significantly inhibited SARS-CoV-2 infection in Vero cells (EC_50_ = 0.08 μM) compared to RPI-28 (**152**) (EC_50_ = 1.2 μM) [161].

Also, carrageenans (CGNs), a group of sulfated D-series polysaccharides with α-galactose residues and possessing negatively charged sulfate ester groups are extracted from marine seaweeds. Thus, carrageenans can interact with the positively charged membrane of SARS-CoV-2 and inhibit entry through the nasal cavity [162]. Morokutti-Kurz et al. [162] tested different SPs for their ability to inhibit viral entry and attachment in SARS-CoV-2 Spike pseudotyped lentivirus (SSPL). They found that ι-carrageenan (**153**) inhibited SSPL cell entry in a dose-dependent manner (IC_50_ = 5.3 μM), whereas at 10 μg/mL, ι-carrageenan (**153**) exerted 80% inhibitory activity against SSPL, in which 100 μg/mL of κ- and λ-carrageenan (**154**) (**155**) were needed each to achieve similar inhibitory activity. Further analysis corroborated the inhibitory effect of ι-carrageenan **153** (3.75 μM) in Vero B4 cells against wild-type SARS-CoV-2 _PR-1_ [162]. A carrageenan-based anti-SARS-CoV-2 nasal spray is currently the subject of clinical trials in the USA, and related studies are also being conducted in the UK [163].

Glycosaminoglycans are another class of sulfated polysaccharides with potential SARS-CoV-2 inhibitory properties. Song et al. [164] checked the inhibitory properties of sulfated glycosaminoglycans (SCSP) (**156**) isolated from sea cucumber *Stichopus japonicus* and observed that SCSP exhibited the highest inhibitory activity (IC_50_ of 9.10 μg/mL) compared to fucoidan from brown algae, and chondroitin sulfate C from sharks (CS). They further demonstrated that SCSP can bind specifically to the S glycoprotein to inhibit entry of SARS-CoV-2 into host cells using pseudotype virus with S glycoprotein of SARS-CoV-2. The authors postulated that the binding of SCSP was facilitated by the high structural flexibility. Flexibility is necessary for the binding of polysaccharides to the S glycoprotein [164,165]. Another sulfated glycosaminoglycan (**156**) from the bacteria, *Pseudomonas* sp. was reported to have a high binding energy with Mpro at −7.98 kcal/mol in silico [5].

In an in vitro study, Jang et al. [166] reported that λ-CGN (**154**) purified from marine red algae suppressed cell entry of SARS-CoV-2 glycoproteins-derived pseudoviruses in a dose-dependent manner. Furthermore, Yim et al. [167] reported virus entry of SARS-CoV-2 was inhibited by crude sulfated fucoidan extracts (**85**) obtained from different seaweeds including *Undaria pinnatifida* sporophyll, *Laminaria japonica*, *Hizikia fusiforme*, *Sargassum horneri*, *Codium fragile*, and *Porphyra tenera*, and *Haliotis discus hannai* [167].

b. Non-sulfated polysaccharides

Aside from sulfated polysaccharides, marine algae also contains other glucans, such as laminarin and alginate β-glucan, which are non-sulfated. A molecular docking and dynamics analysis showed that Laminarin (**157**) has a high binding affinity to the SARS-CoV-2s-glycoprotein and Mpro (−7.83 and −7.81 kcal/mol, respectively). On the other hand, β-glucan (**158**) also exhibited a strong binding affinity towards Mpro (−7.83 kcal/mol) [160]. The brown seaweed species *Laminaria hyperborea*, *Laminaria digitata*, *Macrocystis pyrifera*, and *Ascophyllum nodosum* from the class *Phaeophyceae*, can be used to extract the linear polysaccharide alginates [168]. With regards to alginate, Cano-Vincent et al. [169] previously demonstrated that calcium alginate (**159**) biomaterial can inactivate enveloped viruses such as bacteriophage phi 6 (94.92% viral inactivation) and SARS-CoV-2 Delta variant (96.94% viral inactivation). The authors opine that the negatively charged groups of calcium alginate (**159**) facilitate binding to viral envelopes [5]. In addition, You et al. [170] identified a novel polysaccharide, CLSP-2 (**160**) from edible seaweed *C. lentillifera*, which significantly inhibited SARS-CoV-2 infection in HeLa cells at ≥12.5 μg/mL with an IC_50_ of 48.48 μg/mL.

#### 5.1.2. Proteins

a. Peptides

Bioactive peptides, arising from the hydrolysis of proteins, possess unique amino acid sequences that confer on them various biological activities. Marine organisms are a cheap source of proteins for acquiring bioactive peptides. Yao et al. [171] reported that oligopeptides (2–8 amino acids long) (**161**) arising from in silico hydrolysis of proteins from salmon, squid, tuna, mackerel, and pomfret exhibited high binding affinity to SARS-CoV-2 Mpro and monoamine oxidase A. Peptides that interrupt the binding of SARS-CoV-2 spike proteins to ACE are particularly enticing candidates against SARS-CoV-2 cell entry. For instance, peptides (sequences GDLGKTTTVSNWSPPKYKDTP (**162**) and VW (**163**)*)* obtained from *Thunnus obesus* and *Undaria pinnatifida* have been shown to stably bind to both hACE2 (−246.50 and −117.65 kcal/mol) and spike RBD-ACE2 complex (−223.60 and −123.42 kcal/mole) [23]. The binding of these peptides may disrupt the interaction of SARS-CoV-s2 spike proteins with ACE2 and thus prevent cell entry of the virus. Also, peptides (Asp-Trp (**164**) and Val-Tyr (**165**)) isolated from tilapia viscera hydrolysate exhibited great binding affinity to four SARS-CoV-2 components including Mpro, S-glycoprotein, RBD-ACE2, and deubiquitinase inhibitors [172].

Furthermore, plitidepsin (dehydrodidemnin B) (**166**), a cyclic depsipeptide originally isolated from the tunicate *Aplidium albicans*, is reported to exhibit up to 90% inhibitory activity (at 0.88 nM) against SARS-CoV-2, which is 27.5 times higher compared to remdesivir [173]. Further in vivo studies with mouse models infected with SARS-CoV-2 show that plitidepsin (**166**) reduced the viral load significantly. Also, Ding et al. [174] reported that cyclic dipeptides (**167**) isolated from *Aspergillus versicolor* DY180635, an endophyte of the sea crab (*Chiromantes haematocheir*), exhibited good binding affinity to SARS-CoV-2 Mpro.

Similarly, didemnins, another category of cyclic depsipeptides, can bind to Mpro [175]. For instance, didemnins A, B, and C (**168**–**170**), isolated from Caribbean tunicate (*Trididemnum solidum*) showed high binding affinity (-11.82 kcal/mol, -10.27 kcal/mol, and -9.26 kcal/mol, respectively) to SARS-CoV-2 Mpro [175]. The authors reported that didemnin B (**169**) interacted with the active site of Mpro through hydrogen bonding with the Glu166, an important residue necessary for the hydrolytic activity of the enzyme. The cyclotheonamide peptides, pseudotheonamide C (**171**), and D (**172**) isolated from the marine sponge, *Theonella swinhoei*, likewise were able to bind to the serine protease (TMPRSS2) with binding energies of −11.6 kcal/mol and −10.7 kcal/mol, respectively [134]. Additionally, a modified depsipeptide gallinamide A (also known as symplostatin 4) (**173**) has also been shown to have SARS-CoV-2 inhibitory properties [176]. Isolated from the marine cyanobacteria of the *Schizothrix* genus, gallinamide A (**173**) inhibited cathepsin L, a lysosomal cysteine protease with an EC_50_ of 28 nM, and decreased viral load in VeroE6 cells, with an IC_90_ of 88 nM [176].

b. Lectins

Lectins from marine organisms have been garnering interest lately, especially those from algae [158]. Lectins are carbohydrate-binding non-immunoglobulin-type proteins that recognize specific sugar groups on other molecules [158,177]. Compared to lectins from other sources, marine lectins recognize and bind to a wide variety of sugar moieties including sugar monomers and oligosaccharides [149,158]. Considering that SARS-CoV-2 uses spike glycoproteins to bind and facilitate on the cell surface glycans of potential hosts to initiate entry into cells, this makes them a perfect target for lectins.

Griffithsin (**81**) is a lectin found in the red-algae *Griffithsia* sp., which has a strong specificity for mannose residues of viral glycol proteins [149,158,178]. They have the potential to interrupt the self-assembly of viruses during replication. It has been reported that treatment of SARS-CoV-2-infected rats with 10 mg/kg (b.w.)/day of Griffithsin (**81**) resulted in a 100% survival rate compared to the nontreated group [149,158,178]. Griffithsin (**81**) has been shown to inhibit the s-protein-mediated adhesion of the RBD to hACE2 with an IC_50_ of 0.3 μM [179]. Consequently, Griffithsin (**81**) significantly inhibited SARS-CoV-2 pseudovirus infection in a dose-dependent manner in vitro, with an IC_50_ of 293 nmol/L. Treatment of cells with Griffithsin (**81**) before or at the early stages of infection (0–0.5 h) resulted in up to 80% inhibition of SARS-CoV-2 compared to 32% inhibition when administered 8 h after infection [179]. This suggests that Griffithsin (**81**) is effective against the virus at the initial stages of infection.

c. Protein-bound pigments

Some studies have also highlighted protein-bound pigments as potential inhibitors of SARS-CoV-2 infection. Phycobilins are light-capturing tetrapyrrole chromophores found in certain cyanobacteria, rhodophytes, chloroplasts of red algae, glaucophytes, and some cryptomonads. In recent times, these molecules have been widely studied for their antioxidant and antiviral activities [149,150]. In silico studies have shown that phycocyanobilins (PCB) (**174**), a group of blue phycobilins, have a high binding energy of −8.6 and −9.3 kcal/mol for PCB-Mpro and PCB-RdRp, respectively [180]. Pendyala et al. [150] further showed that PCB (**174**) can bind to Mpro and PLpro via polar interactions with specific binding pockets of amino acids such as G143 (38.5), N119, S46, and Y54 for Mpro, and D164(C), R166(C), D164(A), and G271(A) for PLpro. Petit et al. [181] also reported that PCB (**174**) obtained from *Arthrospira* sp. also exhibited strong binding affinity to SARS-CoV-2 S-glycoprotein using molecular docking studies. They also reported that both van der Waals attractions and hydrogen bonding contributed to the binding of PCB to spike RBD in silico. The molecular docking studies further revealed that PCB interacted with several amino acid residues of the spike RBD including TYR453, GLN493, TYR495, PHE497, ASN501, TYR505, SER494, GLN498, and GLY496 via different bonds [181].

As with PCB (**174**), in silico analysis predicted that other phycobilins such as phycourobilin (PUB) (**175**), phycoerythrobilin (PEB) (**176**), and phycoviolobilin (PVB) (**177**) can bind to SARS-CoV-2 Mpro and PLpro with similar affinity (between −8.2 and −10.0 kcal/mol) [181,182]. Another pigment that can bind to components of SARS-CoV-2 is C-phycocyanin (**178**), a phycobiliprotein from the blue-green algae *Arthrospira platensis*. Raj et al. [183] reported that C-phycocyanin (**178**) competes with ATP for binding to the active site of nsp-12.

#### 5.1.3. Lipids

Lipids are involved extensively in the life cycle of SARS-CoV-2. They form the basis of host cell and viral membranes and act as the initial point of interaction between the virus and its potential host [158].

The receptor binding domain (RBD) of SARS-CoV-2 has been revealed to have three fatty acid binding pockets (FABP), which are lined by hydrophobic amino acids forming a bent tube that serves as an anchor for free fatty acids (FFA) [184]. Linoleic acid (LA) (**179**), an omega 6 (ω-6) PUFA, has been reported to fit into the FABP and occupy all pockets [184]. The binding of LA to the S protein induces the protein to adopt a stable closed S conformation, resulting in a reduced interaction with ACE2 [184]. Similarly, long-chain omega 3 (ω-3) PUFAs, including docosahexaenoic acid (DHA) (**180**) and eicosapentaenoic acid (EPA) (**181**), bind to the FABP and induce the closed conformation of the spike protein, to an even greater extent than LA (**179**) [155]. ω-3 PUFAs therefore have the potential to interrupt the interaction of ACE2 and RBD, thus reducing viral entry. Additionally, increased intake of ω-3 PUFAs decreases inflammation and coagulation caused by COVID-19 [155].

Another group of natural metabolites with a prominent role in cell–cell interactions are cerebrosides. These metabolites also carry out cell regulation and signal transduction. The molecular docking and dynamics analysis by Zahran et al. [185] revealed that cerebrosides such as A1 (**182**) and C1 (**183**) (from the Korean sponge *Haliclona renier*), LAMA-1 and penicilloside B (**184**) (from the Egyptian *Penicillium chrysogenum*), and asperiamide B (**185**) (from the Chinese-Sea-water-derived fungus *Aspergillus niger*) exhibit binding affinity to hACE2 (−7.1 to −7.6, kcal/mol). Moreover, Tassakka et al. [186] found that FAs/lipids were the prominent metabolites both in ethanolic and ethyl acetate extracts of *Halymenia durvillei*, which inhibited the activity of Mpro.

### 5.2. In Silico Studies of Other Secondary Metabolites (Phytochemicals) with Potential Antiviral and Therapeutic Properties against SARS-CoV-2

#### 5.2.1. Polyphenols

SARS-CoV-1 and 2 infections generate reactive oxygen species (ROS) which are known to cause oxidative damage, inflammation, lung infection, and epithelial tissue degeneration. 3CLpro/Mpro activates the NF-kB-dependent reporter gene which causes ROS generation in the HL-CZ cells, leading to a disruption of the oxidation–reduction processes of the cell [178]. Marine organisms are a rich source of antioxidants and several other secondary metabolites classified as broad-spectrum compounds, which can be used as therapies to manage SARS-CoV-2 infection together with antivirals. Polyphenols such as phloroglucinol oligomers and phlorotannins are a type of tannin found in brown algae and have shown promising antiviral action [181]. In silico analysis showed that the phlorotannin dieckol (**86**) can bind to the Spike RBD of SARS-CoV-2 high affinity (−8.1 kcal/mol) [181]. Aatif et al. [187] also reported that dieckol (**86**) from *Ecklonia cava* exhibited similar binding affinity (−8.326 kcal/mol) towards the RBD of the spike protein. Eckol (**186**) and trifucol (**188**) are other phlorotannins obtained from the brown alga *Ecklonia cava* and *Himanthalia elongate*. Eckol (**186**) has a high binding affinity, in silico, to the Mpro (−8.19 kcal/mol), while trifucol (**187**) binds to both the S-glycoprotein (−7.5 kcal/mol) and the Mpro (−6.3 kcal/mol) [160].

Out of 770 compounds, Gentile et al. [134] reported that dieckol (**86**), 8,8-bieckol (**188**), 6,6-bieckol (**189**), and other phlorotannins were among 17 compounds that were predicted to interact with SARS-CoV-2 Mpro. Heptafuhalol A (**190**), a phlorotannin fuhalol from *Sargassum spinuligerum* also exhibited strong binding affinity towards Mpro (−14.60 kcal/mol) by interacting with some amino acid residues within the protease receptor including Thr24, Ser46, Asn142, Glu166, and Pro168. Furthermore, other fuhalol and phlorthol phlorotannin including phlorethopentafuhalol A (**191**) and B (**192**), pseudopentafuhalol C (**193**), hydroxypentafuhalol A (**194**), pentaphlorethol B (**195**), aeruginosin 98B (**196**), resinoside B (**197**), pentaphlorethol A (**198**)**,** and tunichrome An2 (**199**) also have promising SARS-CoV-2 protease inhibitory potentials. Also, the flavonoids -apigenin-7-O-neohesperidoside (rhoifolin) (**200**), luteolin-7-rutinoside (**201**), and resinoside B (**197**) (from the brown alga *Sargassum spinuligerum*) bind to SARS-CoV-2 Mpro with great affinity (−12.4 kcal/mol) [134]. Others have reported that apigenin (**202**) can interact with Mpro via H-bonds between the aromatic region and residues Leu141, Glu166, and Thr190, as well as π-stacking interaction with Gln189 [134]. Using molecular docking, Vijayaraj et al. [5] also showed that esculetin ethyl ester (**203**), a derivative of phenylpropanoid coumarin, from the marine sponge *Axinella* cf. *corrugata* can bind to ARS-CoV-2 Mpro (−8.42 kcal/mol). Esculetin ethyl ester (**203**) has specifically been shown to effectively inhibit SARS-CoV recombinant 3CLpro/Mpro with an ID_50_ value of 46 µmol L^−1^ [146].

#### 5.2.2. Alkaloids

Since the pentacyclic congener ptilomycalin A was discovered to have antiviral properties, polycyclic guanidine alkaloids (PGAs) have generated much interest. PGAs are found in Poecilosclerida sponges such as Batzella, Crambe, and Ptilocaulis, and some starfishes such as *Fromia monilis* and *Celerina heffernani*. In silico analysis of fifteen structurally divergent PGAs against five different proteins of SARS-CoV-2 by El-Demerdash et al. [27] revealed a superior binding affinity of the pentacyclic guanidinic scaffolds crambescidin- 786 (**204**) and 826 (**205**) to different proteases. Crambescidin 786 (**204**) has exceptionally good binding affinities towards Mpro (–8.05 kcal/mol), nucleocapsid phosphoprotein (−6.49 kcal/mol), and nsp10 (−9.06 kcal/mol). Crambescidin 826 (**205**) showed similar binding affinity against Mpro (−7.99 kcal/mol), in addition to S proteins (−6.95 kcal/mol), and nucleocapsid phosphoprotein (−8.01 kcal/mol).

Zahran et al. [185] carried out molecular docking studies of 15 metabolites selected based on their physicochemical properties to investigate their potential effect against the SARS-CoV-2 targets Mpro, methyltransferase (nsp16), RNA-dependent RNA polymerase, RdRp (nsp12) spike protein, and human ACE2 (hACE2). Among them, the glycoside, tirandamycins analogue isotirandamycin B (**206**) and derivatives tirandamycin A (**207**) and B (**208**) extracted from *Streptomyces* sp. exhibited significant binding to SARS-CoV-2 methyltransferase nsp16/10 (−8.4, −8.5 and -8.3 kcal/mol, respectively), Mpro (−7.8, −7.9 and −7.8 kcal/mol, respectively) and RdRp (−7.6, −8.1 and −7.8 kcal/mol, respectively), demonstrating that they are potential SARS-CoV-2 inhibitors. In addition, alteramide A (**209**), a tetracyclic alkaloid extracted from *Pseudoalteromonas* sp., showed a strong binding to RdRp (−9 kcal/mol), nsp16/10 (−8.2kcal/mol), Mpro (−7.1 kcal/mol), and s-protein (−7.4 kcal/mol).

The in silico study by Khan et al. [188] of five marine compounds showed the alkaloids isofistularin-3 (C_31_H_30_Br_6_N_4_O_1_) (**210**) and chimyl alcohol (1-O-hexadecylglycerol) (C_19_H_40_O) (**211**), isolated from *Desmapsamma anchorata*, exhibit high binding affinity towards Mpro. Specifically, isofistularin-3 (**210**) formed hydrogen and hydrophobic interactions with Thr24, Leu27, His41, Phe140, Cys145, His163, Met165, Pro168, and His172, present in the active site and its surroundings. Aspergicin (**212**), an antibacterial alkaloid obtained by mixed fermentation of two marine-derived mangrove epiphytic *Aspergillus* fungi, likewise showed a strong binding affinity towards hACE2 (−17.66 kcal/mol) [189]. Also, callophysin A (**213**), an indole alkaloid from red alga *Callophycus oppositifolius*, showed significant binding properties towards Mpro (−8.776 kcal/mol) [190].

Caulerpin (**214**) is another low toxic bis-indole alkaloid found in distinct species of marine algae, especially the *Caulpera* genus. It has been isolated from the green macroalgae (*Caulerpa racemose*), the red algae (*Chondria armata*), and the brown algae (*Sargassum platycarpum*). Caulerpin (**214**) and some of its derivatives are known to possess a lot of biological properties. Ahmed et al. [147] carried out a molecular docking analysis of caulerpin (**214**) and its analogs against the SARS-CoV-2 Mpro and spike protein. They showed that the derivatives had a higher binding affinity towards Mpro and s-protein than chemical drugs like lopinavir, simeprevir, hydroxychloroquine, chloroquine, and amprenavir.

Abdelrheem et al. [191] also showed that caulerpin (**214**) can bind the SARS-CoV-2 Mpro high affinity (–9.28 kcal/mol) by forming three H-bond interactions with LYS 137, GLU 288, and LYS 5 of 6LU7 amino acid residues. Gaudêncio and Pereira, [192] also reported that benzo[f]pyrano [4,3-b]chromene, notoamide I (a prenylated indole alkaloid from *Aspergillus* sp.) (**215**), emindole SB beta-mannoside (an indole diterpene from *Dichotomomyces cejpii) (***216**), and two bromoindole derivatives, bromodeoxytopsentin (**217**) and dibromodeoxytopsentin (**218**) (which are bisindole alkaloids), can bind SARS-CoV-2 Mpro (binding energies; −8.4, −8.4, −8.2, −7.6 and 7.6 kcal/mol, respectively). Also, 14-debromoaraplysillin I (**219**) (from the marine sponge *Psammaplysilla purpurea*), a phenethylamine monoamine alkaloid, had a binding affinity of −111.52 against RdRp [193].

#### 5.2.3. Terpenes

Ilimaquinone (**220**), a prenylquinone terpene and a member of the monohydroxy-1,4-benzoquinones isolated from the marine sponge *Hippospongia metachromia*, exhibited promising affinity towards active binding pockets of major SARS-CoV-2 proteins including 3CLpro/Mpro (−7.1 kcal/mol), 6M0J (−6.9 kcal/mol), PLpro (- 8.1 kcal/mol), Nsp10 (−7.6 kcal/mol), Nsp14 (−8.1 kcal/mol), and Nsp13 (−8.2 kcal/mol) [154]. Sepay et al. [194] screened more than fifty natural products from various sources for their binding potentials towards SARS-CoV-2 and reported that the terpenoid (T3) (**221**) from marine sponge *Cacospongia mycofijiensis* showed the best binding score at −9.1 kcal/mol. Also, the terpenoids T1 (**222**), T2 (**223**), and T4 (**224**), which are all geometric T3 (**222**), showed good binding scores as well (−8.6, −8.20, and −8.08 kcal/mol, respectively). The author postulated that the higher hydrophobicity and lower flexibility of these terpenoids augmented their affinities towards SARS-CoV-2 Mpro.

Dictyosphaeric acid A (**225**) (a polyketide decalactone from the green alga *Dictyosphaeria versluyii)* and excavatolide M (**226**) (a briarane-type diterpene from the coral *Briareum excavatum)* showed inhibitory activities against SARS-CoV-2 by disrupting the interaction of TMPRSS2-SPPIs [195]. However, the drug ability test showed that excavatolide M (**226**) is toxic and not suitable for use as a drug [195]. The diterpenoid, hamigeran b (**227**) from the marine sponge *Hamigera tarangaensis*, also showed high binding affinity towards Mpro (−7.98 kcal/mol) [5]. Also, the terpenoid fasciospongide A (**228**) (from the sponge *Fasciospongia* sp.) and epolactaene (**229**) (from the fungus *Penicillium* sp.) have been shown to bind to 3CLpro/Mpro with high affinity, −104.37 and −102.9 kJ/mol, respectively [193]. The terpenoid steroid moniloside A (**230**) (from the starfish *Formia monilis*) also showed a strong binding affinity (−63.12 kJ/mol) toward RdRp [193].

#### 5.2.4. Other Metabolites

The phytocompounds of *Corallina officinalis*, *Caulerpa racemos*, *Colpomenia sinuosa*, *Gracilaria edulis Gracilaria corticata*, *Sargassum wightii*, and *Ulva fasciata* were studied for their numerous antiviral metabolites as potential inhibitors of SARS-CoV-2 ACE2-bound omicron B.1.1.529 spike protein trimer [196]. The molecular docking analysis showed that the glycoside, caffeic acid hexoside (**231**), and phloretin (**232**) from *S. wightii* can bind to crucial residues ASN417, SER496, TYR501, and HIS505 of spike protein, which support angiotensin-converting enzyme II receptor interaction [196]. Also, the lipid sterol cholestan-3-ol, 2-methylene-, (3beta, 5 alpha) (CMBA) (**233**) from *C. officinalis* showed a strong binding potential (−6.0 kcal) towards the omicron RBD mutated residues LEU452 and ALA484.

ACE2 produced by marine organisms can also serve as a receptor binding domain to the SARS-CoV-2 spike glycoprotein to suppress its transmission. For instance, ACE2 (**234**) of *Delphinapterus leucas* (Beluga whale) had a binding affinity of −988.5 kcal/mol towards the SARS-CoV-2 spike glycoprotein which is comparable to the hACE2 binding affinity (−946.4 kcal/mol) to the spike glycoprotein. Thus, ACE2 and ACE2-like structures from the marine biota could be used as decoys for viral binding [197]. Fayed et al. [198] screened several marine compounds for their pharmacophore potentials against SARS-CoV-2 Mpro (6lu7 and 6y2f), spike glycoprotein, and RNA Polymerase, and reported that compounds with a flavonoid core, acyl indole, and pyrrole carboxamide alkaloids performed better. The co-crystallized ligands of Mpro showed perfect overlay with the pyrroles sceptrin (**235**) and debromo sceptrin (**237**). Among all the target proteins, thalassiolin (A-C) (**237***–***239**) had the best binding and similarity values. Also, ACE2 and Mpro were shown to interact well with compounds isolated from marine sponges including microspinosamide (**240**) (−16.8 and 13.7 kcal/mol), neamphamide A (**241**) (−13.7 and 13.1 kcal/mol), mirabamide A (**242**) (−11.3 and 10.3 kcal/mol), and sterol clathsterol (**243**) (−10.5 and 10.1 kcal/mol) [199]. However, the drug-likeness test of all compounds was below Lipinski’s rule of 5, even though all the compounds had shown potential inhibition against the HIV-1 virus.

Structurally, the natural inorganic polyphosphate (polyP), considered a physiological, metabolic energy (ATP)-providing and morphogenetically active linear polymer of orthophosphate released from human blood platelets, is expressed in every cell including marine bacteria and sponges [200,201]. Polyphosphate (polyP) helps in the mediation of blood clots due to interaction with the protease coagulation factor VII; however, its production is reduced in COVID-19 patients due to a deficiency in platelet count [201]. Müller et al. [202] and Neufurth et al. [200] found that polyp (**244**) blocks the binding of the receptor binding domain (RBD), thus preventing the binding of the spike protein to host ACE-2 receptor at concentrations ranging from 1 to 100 µg/mL with 70% effectiveness at 10 µg/mL. Neufurth et al. [200] proposed that the 15 phosphate units of polyP (**244**) interacted with the basic residues, Arg, Lys, and His on the spike protein. Müller et al. [202] also reported polyp (**244**) increased ATP production, cell attachment, and expression of the membrane-tethered mucin MUC1 and the secreted mucin MUC5AC genes in the mucus layer, thus enhancing the barrier against inhaled pathogens such as the coronavirus SARS-CoV-2 [202,203].

**Figure 4:**
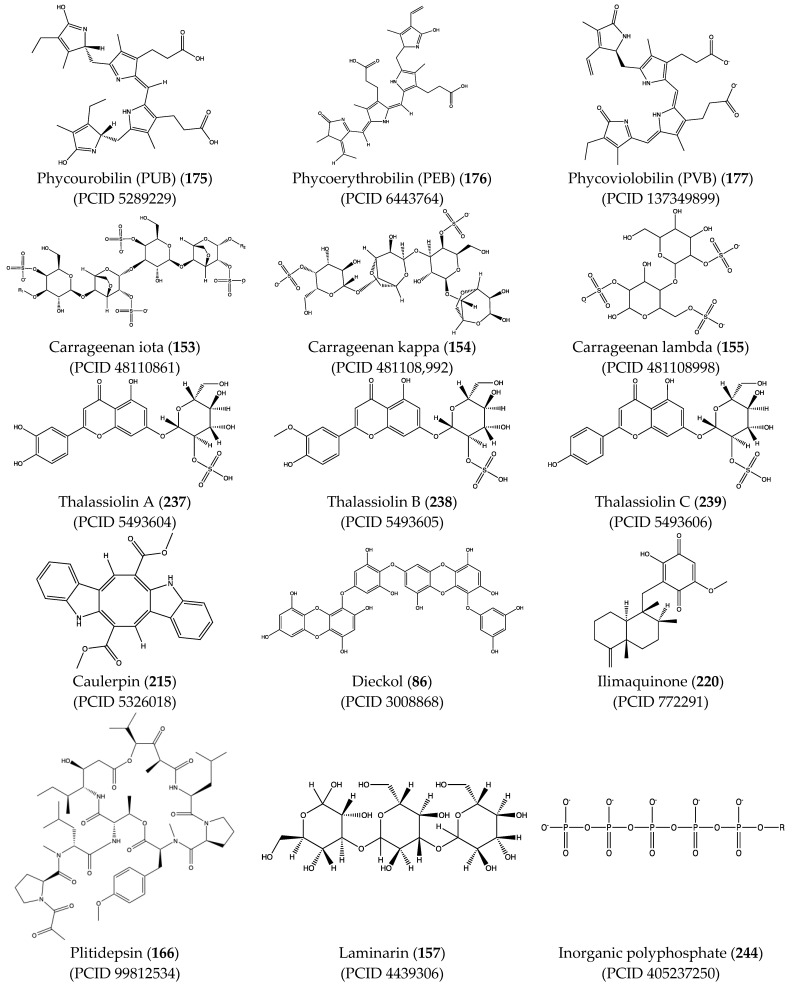
Chemical structure of some selected compounds from marine organisms with SARS-CoV-2 inhibitory properties.

Structural characteristics of some selected compounds from marine organisms with SARS-CoV-2 inhibitory properties are shown in Figure 4 and Figure S8. Additionally, Table S1 shows some selected marine compounds with potential inhibitory properties against SARS-CoV-2 in silico.

## 6. A Promising Future for Marine Bioactive Metabolites to Tackle SARS-CoV-2

Due to the vast and diverse organisms with naturally occurring metabolites found in bodies of water, researchers are increasingly turning to the oceans, rivers, and seas for new natural compounds with antiviral potentials to help create the basis for novel therapeutics. Compounds of various structural classes, including polysaccharides, terpenes, steroids, alkaloids, and peptides that inhibit both RNA and DNA viruses have been isolated from marine micro- and macro-organisms. There is much hope to discover novel resources from marine organisms, which would serve as potential drug leads that would, in addition to controlling viral replication, help manage the symptoms presented by viral diseases. Such compounds could either block the penetration of viruses into the host cells, inhibit viral fusion to host proteins, or inhibit the activity of major viral proteins such as those involved in replication. However, drug development is cost-intensive. Even with significant efforts being made in designing novel SARS-CoV-2 inhibitors from marine organisms, with some (such as plitidepsin (**166**)) already under clinical trials, most available studies appear exceptionally preliminary and based on computer-aided findings that employed molecular docking, molecular dynamics simulation techniques, and network pharmacology. The scarcity of comprehensive pre-clinical research involving various cell lines and animal models underscores the need for in-depth future investigations. Such studies should examine the compounds identified through in silico analysis for their potential drug-like properties, laying the groundwork for subsequent clinical trials.

## Figures and Tables

**Table 1:** Antiviral properties of metabolites from marine bacteria.

Group of Compounds	Compound Name and Structural Formula (PubChem CID)	Marine Microorganism(s)	Source of Microorganism(s)	Antiviral Activity	Refs.
Alkaloid	9(10H) acridanone (Acridone) (**9**) (2015) 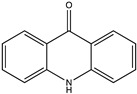	*Streptomyces fradiae* VITMK2	Marine soil sediment collected from mangrove forest region of Pichavaram Tamil Nadu, India	Antiviral activity against WSSV in shrimps with a survival rate of 88.9%, 83.3%, and 55.6% at doses of 500 μg, 250 μg, and 125 μg/animal	[37]
Butenolide	(4S)-10-hydroxy-10- methyl-11-oxo-dodec-2-en-1,4-olide (Avenolide) (**10**) (129320493) 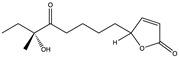	*Streptomyces* *koyangensis* SCSIO 5802	Isolated from the south China sea	Antiviral activity against HSV (EC_50_ 25.4 μM)	[38]
Amide (diketopiperazines)	(3Z,6Z)-3-(4-hydroxybenzylidene)-6-isobutylidenepiperazine-2,5-dione (**11**) (135034789) 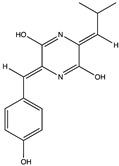	*Streptomyces* sp. FXJ7.328	Coastal sediment collected at Huanghai Beach, Dalian, China	Anti-H1N1 activity at IC_50_ of 41.5 ± 4.5 μM	[39]
Polyketide abyssomicin	Neoabyssomicin D (**12**) (139590101) 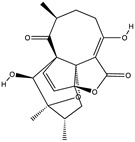	*Streptomyces* *koyangensis* SCSIO5802	Sediment sample collected from the South China Sea	Antiviral activity against HSV at a concentration of 10 μM	[40]

WSSV (white spot syndrome virus), HSV (herpes simplex virus), H1N1 (a subtype of influenza A virus), half maximal effective concentration (EC_50_); half maximal inhibitory concentration (IC_50_).

**Table 2:** Antiviral activities of marine-derived bioactive compounds extracted from marine fungi.

Group of Compounds	Compound Name and Structural Formula (PubChem CID)	Marine Microorganism(s)	Source of Microorganism(s)	Antiviral Activity	Refs.
Alkaloid	Trichobotrysin A (**27**) (132594639) 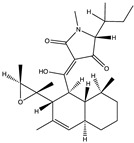 Trichobotrysin B (**28**) (132594640) 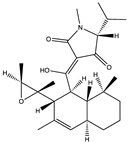 Trichobotrysin D (**29**) (132594642) 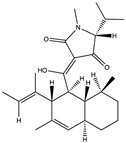	*Trichobotrys effuse* DFFSCS021	Deep sea sediment collected from the South China Sea	Anti-HSV-1 activity at TC_0_ with IC_50_ values of 3.1, 9.4, and 3.1 μM	[51]
Fumiquinazoline Alkaloid	Neosartoryadin A (**30**) (132552299) 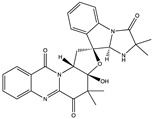 Neosartoryadin B (**31**) (132552300) 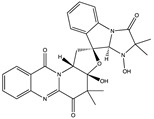	*Neosartorya udagawae* HDN13-313	Isolated from the root of the mangrove plant *Avicennia marina*	Anti-H1N1 activity with IC_50_ values of 66 and 58 μM	[52]
Indole diketopiperazine alkaloid	Raistrickindole A (**32**) (145720909) 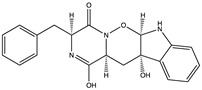 Raistrickin (**33**) (135666745) 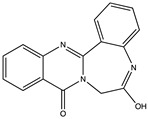	*Penicilliumraistrickii* IMB17-034	Marine sediment collected from mangrove swamp	Anti-HCV activity at EC_50_ values of 5.7 and 7.0 μM (against VX-950 positive 0.05 μM control) and CC_50_ values of >200 μM	[53]
	Trypilepyrazinol (**34**) (146682634) 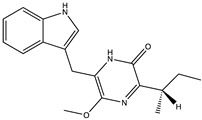	*Penicillium* sp. IMB17-046	Marine sediments collected from a mangrove swamp in Sanya, Hainan province, China	Anti-HIV and anti-HCV activities with IC_50_ values of 4.6 and 7.7 μM and CC_50_ values of 44.3 and 116.1 μM, respectively	[54]
Quinone	Aspergilol H (**35**) (137655878) 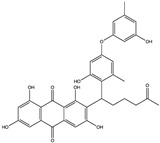 Aspergilol I (**36**) (137640339) 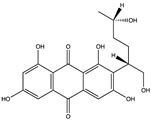 Coccoquinone A (**37**) (132512004) 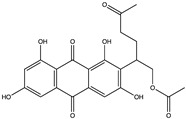	*Aspergillus* *versicolor* SCSIO41502	Deep sea sediment collected from the South China Sea	Anti-HSV-1 activity at EC_50_ values of 4.7, 6.3, and 3.1 µM and CC_50_ values of 108.6, and 50.7 µM, respectively	[55]
	Seco-penicitrinol A (**38**) (146683966) 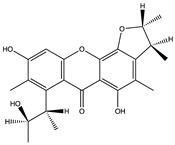	*Aspergillus sydowii* EN-534 and *Penicillium citrinum* EN-535	Isolated from the same fresh tissue of the marine red alga *Laurencia okamurai.*	Inhibitory activity against influenza neuraminidase with an IC_50_ value of 24.7 nM	[56]
	(–)-2′R-1-hydroxyisorhodoptilometrin (**39**) (132279362) 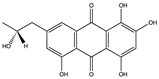 Methyl 6,8-dihydroxy-3-methyl-9-oxo-9H-xanthene-1-carboxylate (**40**) (132278119) 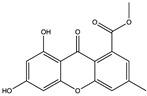	*Penicillium* sp. OUCMDZ-4736	Soil sediment around roots of mangrove plant, *Acanthus ilicifolius*	Anti-hepatitis B virus activity by inhibiting HBsAg and HBeAg secretion in HepG2.2.15 cells (IC_50_ values of 4.6 and 11. 4 µM and no cytotoxicity at 20 µM)	[57]
	(Z)-1-((1-hydroxypenta-2,4-dien-1-yl) oxy) anthracene-9,10-dione (**41**) (134822307) 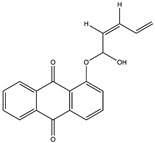	*Nocardia alba* KC710971	Mangrove soil collected from Nellore region of Andhra Pradesh, India	In ovo antiviral activity against two poultry viruses NDV and IBDV, at doses of 25–100 mg	[58,59]
Peptides thiodiketopiper-azine-type alkaloid	Eutypellazines A-L (**42**–**53**) (129909516) (see Figure S1 in Supplementary File)	*Eutypella* sp. MCCC 3A00281	Deep sea sediment collected from the South Atlantic Ocean	Anti-HIV activity with IC_50_ ranging between 1 and 19 µM and CC_50_ > 100 µM	[60]
	Acremonpeptide A (**54**) (145721238) 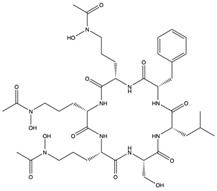 Acremonpeptide B (**55**) (145721239) 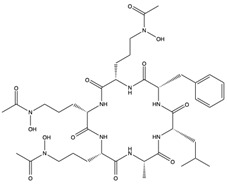 Al(III)-acremonpeptide D (**56**) (145721242) 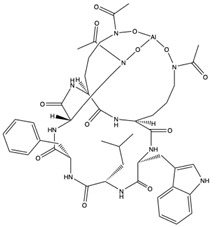	*Acremonium* *persicinum* SCSIO 115	Marine sediment South China Sea	Anti-HSV-1 activity (EC_50_ values of 16, 8.7, and 14 µM)	[61]
	Simplicilliumtide J (**57**) 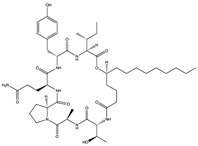 Verlamelin A (**58**) (139588823) 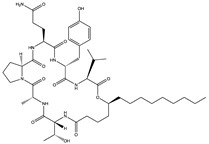 Verlamelin B (**59**) (139588455) 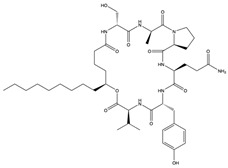	*Simplicillium* *obclavatum* EIODSF 020	Marine sediment samples collected in the East Indian Ocean	Anti-HSV-1 activity against Vero cells (IC_50_ values of 14.0, 16.7, and 15.6 µM), TC_0_ and TC_50_ values of 25.1 and 204 µM for simplicilliumtide J, 57.2 and 137.0 µM for verlamelin A, and 49.4 and 101.1 µM for verlamelin B	[62]
Prenylated indole diketopiperazine	Neoechinulin B (**60**) (23425626) 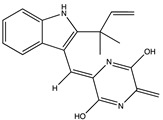	*Eurotium rubrum* F33 (MCCC3A00287)	Sediment collected from 2067 m depth under the South Atlantic Ocean	Anti-influenza A/WSN/33 virus activity (EC_50_ 27.4 μM, CC_50_ > 200 μM)	[63]
Enantiomeric alkaloid dimer	(+)-pestaloxazine A (**61**) (145256980) 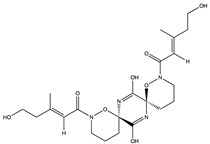	*Pestalotiopsis* sp. (ZJ-2009-7-6)	Derived from a soft coral from the South China Sea	Antiviral activity against EV71 (IC_50_ value of 14.2 ± 1.3 μM compared to ribavirin at IC_50_ 256.1 ± 15.1 μM) and a TC_50_ value of 130.2 ± 10.1 μM	[64]
	Aspergillipeptide D (Aspergillide D) (**62**) (132496356) 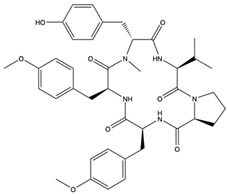 Aspergillipeptide E (Aspergillide E) (**63**) (139591200) 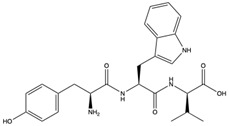	*Aspergillus* sp.	China South Sea Gorgonian *Melitodes squamata.*	Anti-HSV-1 activity with IC_50_ values of 9.5 and 19.8 μM and TC_0_ and TC_50_ values of 81.9 and 204.4 mM for aspergillide D and 153.2 and 346.0 mM for spergillide E	[65]
Polyketone	Pestalotiolide A (**64**) (156581239) 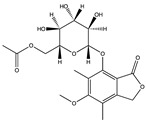	*Pestalotiopsis* sp. (ZJ-2009-7-6)	Soft coral *Sarcophyton* sp. collected from Yongxing Island in the South China Sea	Anti-EV71 activity at IC_50_ of 27.7 μM and TC_50_ value of 254.9 μM (Ribavirin IC_50_ value 418.0 μM)	[66]
	Truncateol M (**65**) (156580490) 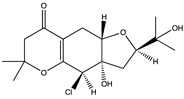	*Truncatella* *angustata*	Finger sponge *Amphimedon* sp. collected from the reef in Yongxing Island in the South China Sea	Anti-H1N1 activity with an IC_50_ value of 8.8 µM and CC_50_ > 100 µM	[67]
	Epiremisporine B (**66**) (139584953) 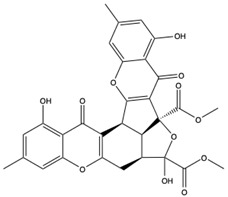	*Penicillium* sp. SCSIO	Deep sea sediment	Anti-EV71 activity (IC_50_ = 19.8 µM)	[68]
	Spiromastilactone D (**67**) (127026306) 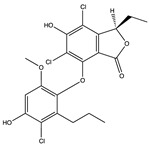	*Spiromastix* sp. MCCC3A00308	Deep sea sediment from South Atlantic Ocean	Antiviral activity against WSN influenza virus (IC_50_ = 6.0 µM, CC_50_ > 100 µM)	[69]
Pyrone	Methyl-(4-chloro-l,6-dihydroxy-3-methylxanthone)-8-carboxylate (**68**) (156581832) 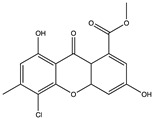	*Aspergillus iizukae* KL33	Coastal saline soil in Kenli, Shandong Province of China	Anti-H1N1 activity (IC_50_ = 44.6 µM) Anti-HSV-1 (IC_50_ = 21.4 µM) Anti-HSV-2 (IC_50_ = 76.7 µM)	[70]
Sterol	3α-hydroxy-pregn-7-ene-6,20-dione (cladosporisteroid B) (**69**) 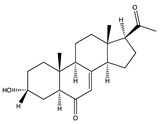	*Cladosporium* sp. WZ-2008-0042 *Cladosporium* sp. SCSIO41007	Gorgonian *Dichotella gemmacea* collected at the Meizhou Island coral reef, from the South China Sea and *Callyspongia* sp. sponge collected from the sea area near Xuwen County, Guangdong Province, China	Antiviral activity against RSV (IC_50_ = 0.12 mM) and H3N2 (IC_50_ = 16.2 μM) and a TC_50_ value of 1.19 mM	[71,72]
	3β-hydroxyergosta- 8,14,24(28)-trien-7-one (**70**) 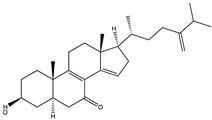	*Penicillium* sp. IMB17-046	Marine sediments collected from a mangrove swamp in Sanya, Hainan province, China	Antiviral activities against HIV (IC_50_ =3.5 µM, CC_50_ = 51.2 ± 3.5 µM) and IAV (IC_50_ = 0.5 µM, CC_50_ > 100 μM)	[54]
Terpenoid	Talaromyolide D (**71**) (146683236) 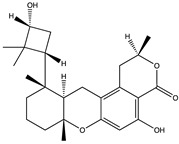	*Talaromyces* sp. CX11	NA	Antiviral activity against pseudorabies virus at a concentration of 1.56–25 μM (CC_50_ =3.4 μM)	[73]
	3-hydroxypentacecilide A (**18**) (154573703) 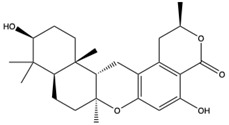 Chrodrimanin N (**17**) (139589677) 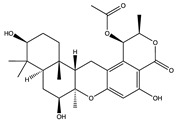	*Penicillium* sp. SCS-KFD09	Marine worm of Haikou Bay, China	Anti-H1N1 activity (IC_50_ values of 34 and 58 µM)	[47]
	Stachybonoid A (**72**) (156581564) 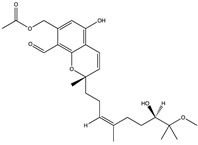	*Stachybotrys chartarum* 952	Crinoid (*Himerometra* *magnipinna*) isolated from Xuwen Coral Reef Nature Reserve, Zhanjiang City, Guangdong Province, China	Inhibitory effect on dengue viral protein (prM) at concentrations of 5–50 µM	[74]
	Stachybogrisephenone B (**73**) (139583964) 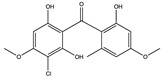	*Stachybotry* sp. HH1 ZSDS1F1-2	Sponge at Xisha Island China	Antiviral activity against EV71 (IC_50_ = 30.1 μM)	[75]
Spirocyclic γ - lactam	Spirostaphylotrichin X (**74**) (153210908) 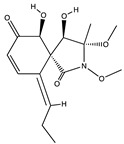	*Cochliobolus* *lunatus* SCSIO41401	Marine alga at Yongxing Island South China Sea	Antiviral activity against influenza virus strains with IC_50_ values from 1.2 to 5.5 µM and CC_50_ > 200 μM	[76]
Xanthone	Norlichexanthone (**75**) (5281657) 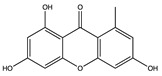 Griseophenone A (**76**) (3083749) 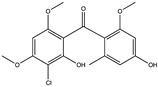	*Stachybotry* sp. HH1 ZSDS1F1-2	Sponge at Xisha Island China	Antiviral activity against EV71 (IC_50_ values of 50.0 and 40.3 μM)	[75]

HSV-1 and 2 (herpes simplex virus type 1 and 2), WSN (H1N1) (a subtype of influenza A virus), HCV (hepatitis C virus), HIV (human immunodeficiency virus), IAV (influenza A virus), EV71 (enterovirus 71), RSV (respiratory syncytial virus), (influenza A virus), NDV (Newcastle disease virus), H3N2 (a subtype of influenza A virus), IBDV (infectious bursal disease), NA (not available), non-cytotoxic concentration (TC_0_), half maximal toxic concentration (TC_50_), half maximal effective concentration (EC_50_), half maximal inhibitory concentration (IC_50_), half maximal cytotoxic concentration (CC_50_).

**Table 3:** Antiviral properties of metabolites from marine macro-organisms.

Group of Compounds	Compound Name and Structural Formula (PubChem CID)	Type of Marine Macroorganism(s)	Marine Macroorganism(s)	Source of Macroorganism(s)	Antiviral Activity	Refs.
Peptides	Bengamide A (**133**) (10077016) 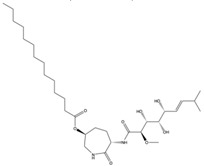	Sponge	*Jaspis* cf. *coriacea*	NA	HIV-1 inhibitor (EC_50_ = 0.015 ± 0.009 μM and CC_50_ = 2.5 ± 1.0 μM)	[92]
	Stellattapeptins A (**134**) and B (**135**) (See Figure S7 in Supplementary File)	Sponge	*Stellatta* sp.	North-western Australia	Anti-HIV-1 activity (EC_50_ of 23 and 27 nM)	[93]
Macrodiolide	Pateamine A (**136**) (10053416) 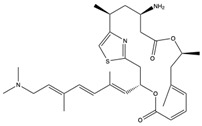	Sponge	*Mycale* sp.	NA	Inhibition of sindbis virus genomic mRNA at concentrations of 50–400 nM	[96]
Nortopsentin alkaloids	Nortopsentin indole-imidazole derivative (**137**) 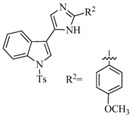	Sponge	*Spongosorites ruetzleri*	Deepwater	Anti-TMV activity (50% and 18% inactivation inhibitory effect in vivo at 500 and 100 μg/mL)	[97]
Alkaloids	Polycarpine bis(2,2,2-trifluoroacetate) (**138**) 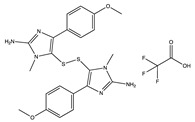	Ascidian	*Polycarpa* *aurata*	NA	Anti-TMV activity (57% and 19% inactivation inhibitory effect in vivo at 500 and 100 μg/ml	[98]
Diterpenoids	Ehrenbergol C (**139**) (163116389) 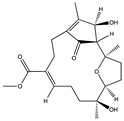 Acetyl ehrenberoxide B (**140**) 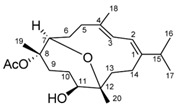	Soft coral	*Sarcophyton* *ehrenbergi*	Collected at San-Hsian-Tai Taitong County, Taiwan	Anti-HCMV activity (EC_50_ of 52.8 and 22 μM)	[99]
Cembrane-type diterpenoids	Secocrassumol (**141**) (129905909) 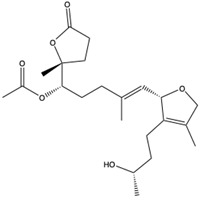	Soft coral	*Lobophytum crassum*	Collected from the coral reefs, at Dongsha Atoll off Taiwan	Anti-HCMV activity (IC_50_ = 12.7 μM)	[100]
Steroids	Echrebsteroid C (**142**) (90680710) 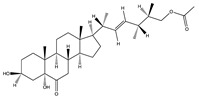	Coral	*Echinogorgia rebekka*	Collected from the South China Sea	Anti-RSV activity (IC_50_ = 0.19 μM; TC_50_ = 24.4 μM)	[101]
Polyhydroxylated steroids	(24R)-methylcholest-7-en-3β,5α,6β-triol (**143**) 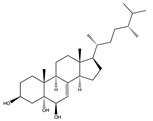 (24S)-ergost-3β,5α,6β, 11α-tetraol (sarcoaldesterol B) (**144**) (10718409) 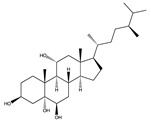	Cnidarian (Soft coral)	*Sarcophyton* sp.	South Sea (Weizhou Islands Sea area)	Anti-H1N1 IAV activity (IC_50_ of 19.6 μg/mL and 81.4 μM)	[102]
Peptide	LvSWD3 (SWD gene) (**145**)	Crustacean (Pacific white shrimp)	*Litopenaeus* *vannamei*,	Shrimp farm in Zhanjiang, Guangdong Province	Anti-WSSV activity at 5 μg/shrimp	[94]
Polysaccharide	Acidic mucopolysaccharide (**146**)	Echinoderm	*Stichopus* *japonicus* selenka	NA	Anti-HBV activity at concentrations of 30–50 mg kg^−1^)	[133]
Enzyme	Phospholipase A2 (AP-PLA-2) (**132**) (See Figure S6 in Supplementary File)	Echinoderm (starfish)	*Acanthaster planci*	Moluccas Islands, eastern Indonesia	Anti-HIV1 activity (LC_50_ of 1.6 mg/mL)	[105]
Naphthopyrones	Comaparvin (**147**) (324099) 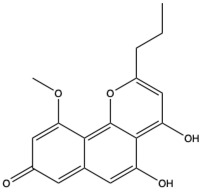	Echinoderms (crinoids)	*Capillaster multiradiatus*	Collected from the Torres Strait, Queensland, Australia	Anti-HIV-1 activity with EC_50_ of 7.5 ± 1.7 µm	[106]
Proteins/ peptides	Papain hydrolysate (>100 kDa fraction) (**148**)	Echinoderms (sea cucumbers)	aquapharyngeal bulb of *Cucumaria frondosa*	Collected in Passamaquoddy Bay, Bay of Fundy, New Brunswick, Canada	Anti-HSV-1 activity (EC_50_ of 18.2 µg/mL; CC_50_ > 500 µg/mL)	[95]
Sulfated steroids	Disodium 2β,3α-dihydroxy-6E-hydroximine-5α-cholestane-2,3-disulfate (**149**) 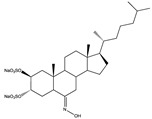	Echinoderms	Echinoderms from cold waters	South Atlantic Ocean	Antiviral activity against HSV-1 (EC_50_ = 16.5 ± 1.4 µg/mL; CC_50_ > 100 µg/mL)	[103]
Polysaccharide	Water-soluble polysaccharides (**150**)	Worm	*Sipunculus nudus*	Collected from the Xiamen market, China	Inhibition of the HBV DNA and HBsAg mRNA synthesis at concentrations of 1, 0.5, 0.25, and 0.13 mg/mL)	[104]

HSV-1 (herpes simplex virus type 1), WSN (H1N1) (subtype of influenza A virus), HBV (hepatitis B virus), HIV (human immunodeficiency virus), WSSV (white spot syndrome virus), TMV (tobacco mosaic virus), HCMV (human cytomegalovirus), RSV (respiratory syncytial virus), NA (not available), half-maximal lethal concentration (LC_50_), half maximal toxic concentration (TC_50_), half maximal effective concentration (EC_50_), half maximal inhibitory concentration (IC_50_), half maximal cytotoxic concentration (CC_50_).

## Data Availability

Not applicable.

## Supplementary Materials

The following supporting information can be downloaded at: https://www.mdpi.com/article/10.3390/ph17030328/s1, Figure S1: Chemical structures of identified compounds from marine microorganisms (Bacteria and fungi) that inhibit viruses; Figure S2: Eutypellazines A-L isolated from Eutypella sp. found in deep sea sediment collected from the South Atlantic Ocean; Figure S3: Chemical structures of identified compounds from marine microorganisms (marine algae) that inhibit viruses; Figure S4: Chemical structures of identified compounds from marine plants that inhibit viruses; Figure S5: Chemical structures of identified compounds from marine macro-organisms (invertebrates) that inhibit viruses; Figure S6: Phospholipase A2 (AP-PLA-2) from Echinoderm (starfish) found in Moluccas Islands, eastern Indonesia; Figure S7: Stellattapeptins A and B isolated from Stellatta sp. found in North-western Australia; Figure S8: Chemical structures of identified compounds from marine organisms with inhibitory properties against SARS-CoV-2; Table S1. Selected marine compounds with potential inhibitory properties against SARS-CoV-2 in silico. References [5,60,93,105,134,144,146,150,175,180,181,188,195,198] are cited in the Supplementary Materials.
