# Supplementary material for: Marine-Derived Bioactive Metabolites as a Potential Therapeutic Intervention in Managing Viral Diseases: Insights from the SARS-CoV-2 In Silico and Pre-Clinical Studies
Source: Pharmaceuticals (Basel). 2024 Mar 1;17(3):328. doi: 10.3390/ph17030328 (PMC10975954; doi:10.3390/ph17030328)
Supplement: Supplementary file 1 [file pharmaceuticals-17-00328-s001.zip › pharmaceuticals-2852677-supplementary.pdf]

Supplementary file for Marine-Derived Bioactive Metabolites as a Potential Therapeutic Intervention in Managing Viral Diseases: Insights from the SARS-CoV-2 In Silico and Pre-Clinical Studies

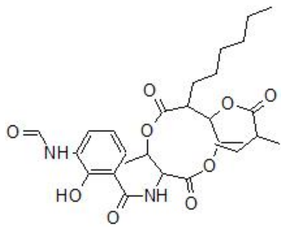

Antimycin A1a (**1**)
(23629646)

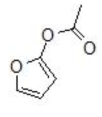

Furan-2-yl acetate (**2**)
12719907

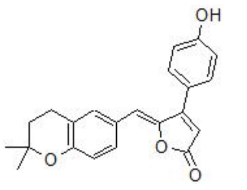

Rubrolide S (**13**)
101885283

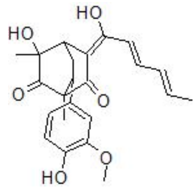

Sorbicatechol (**14**)
A 76310269

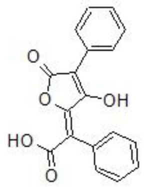

Pulvic acid (**15**)
54682513

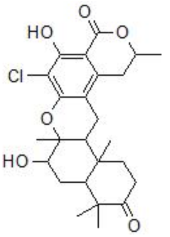

Chrodrimanin K (**16**)
132967434

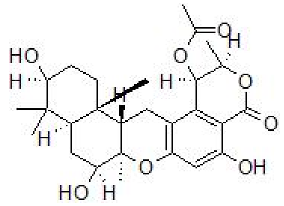

Chrodrimanin N (**17**)
139589677

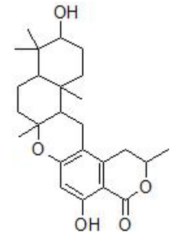

3-hydroxypentacecilide A (**18**)
154573703

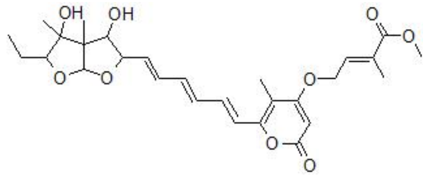

Asteltoxin E (**19**)
156581380

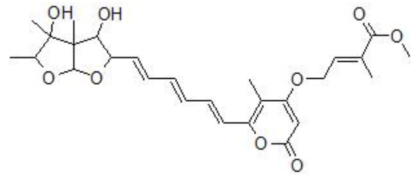

Asteltoxin F (**20**)
156581381

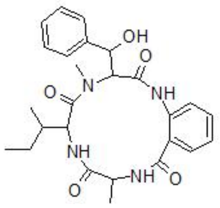

Asperterrestide A (**26**)
71726177

Figure S1. Chemical structures of identified compounds from marine microorganisms (Bacteria and fungi) that inhibit viruses.

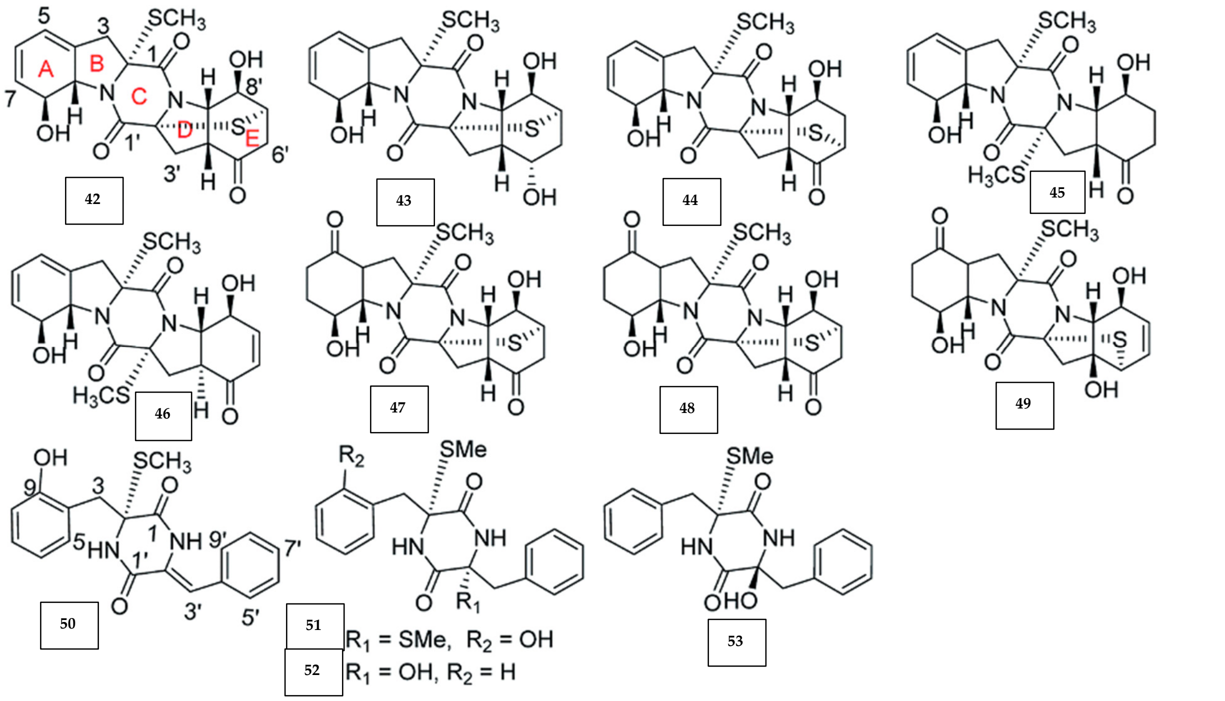

Figure S2. Eutypellazines A-L **42-53** found in deep sea sediment collected from the south Atlantic Ocean [1].

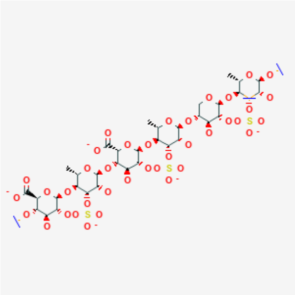

Ulvan (**84**)
405234592

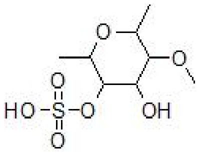

Fucoidan (**85**)
129532628

Figure S3. Chemical structures of identified compounds from marine microorganisms (marine algae) that inhibit viruses.

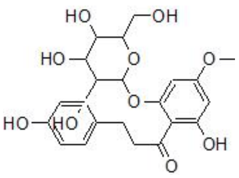

Asebotin (**84**)
11190157

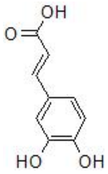

Trans-caffeic acid (**88**)
689043

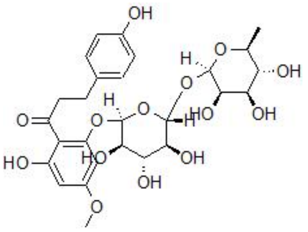

Thalassodendrone (**90**)

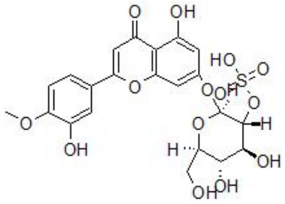

Thalassiolin D (**91**)

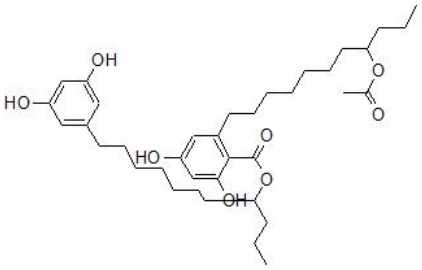

Integracin A (**92**)
486005

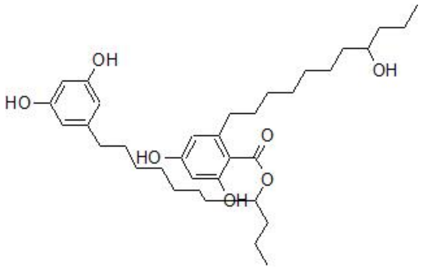

Integracin B (**93**)
70678748

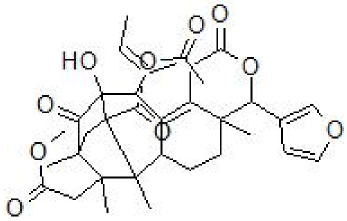

Thaixylomolin I (**94**)
355351742

Figure S4. Chemical structures of identified compounds from marine plants that inhibit viruses.

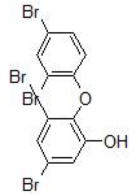
3,5-dibromo-2-(2,4-dibromo-
phenoxy)-phenol (**95**)
3086109

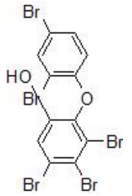
3,4,5-tribromo-2-(2,4-
dibromophenoxy)-
phenol) (**96**)
11952901

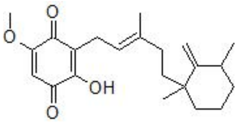
Metachromin A (**97**)
10473589

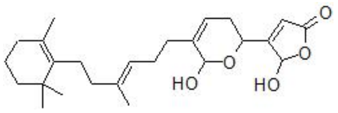
Manoalide (**98**)
6437368

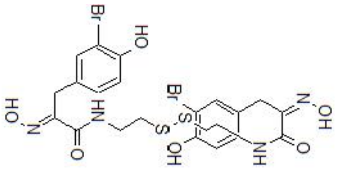
Psammaplin A (**99**)
6400741

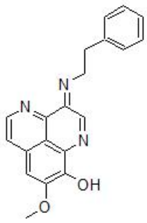
3-(phenethyla-
mino)deme-
thyl(oxy)aaptamine
(**100**)
136008231

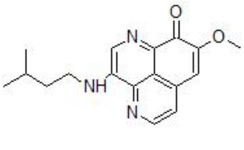
3-(isopentylamino)de-
methyl(oxy)aaptamine
(**101**)

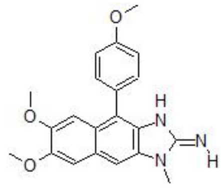
Kealiinine B (**102**)
23248790

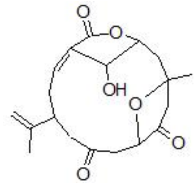
5-episinuleptolide (**113**)
101155443

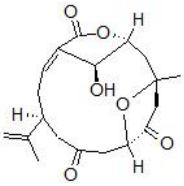
Sinuleptolide (**114**)
6442255

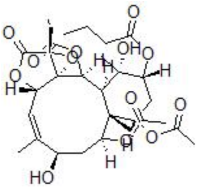
Briacavatolide C (**115**)

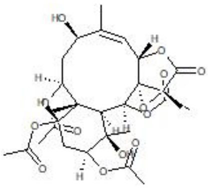
Briacavatolide F (**116**)

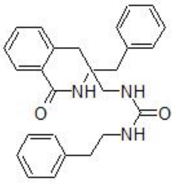
Molleurea A (**125**)
66553500

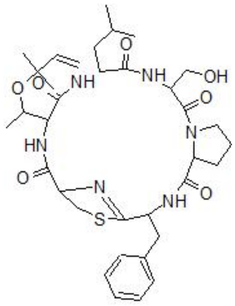
Mollamide E (**126**)
66553498

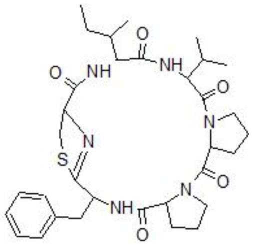
Mollamide F (**127**)
70697324

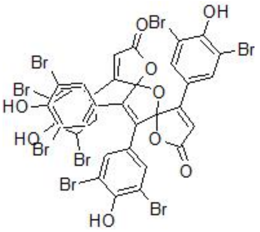
Prunolide A (**128**)
21589554

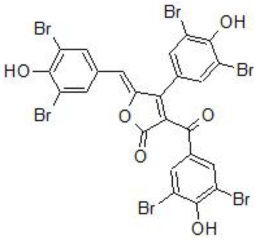
Cadiolide B (**129**)
11400288

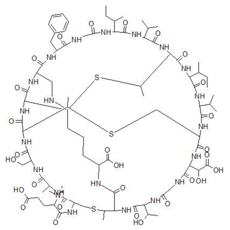
Divamide A (**130**)
131801640

Figure S5. Chemical structures of identified compounds from marine macro-organisms (invertebrates) that inhibit viruses.

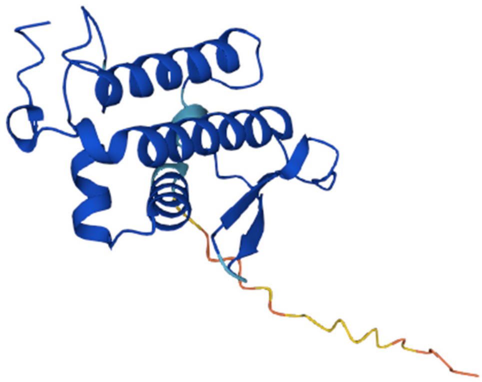

Figure S6. Phospholipase A2 (AP-PLA-2) (**132)** from Echinoderm (starfish) found in Moluccas Islands, eastern Indonesia [3].

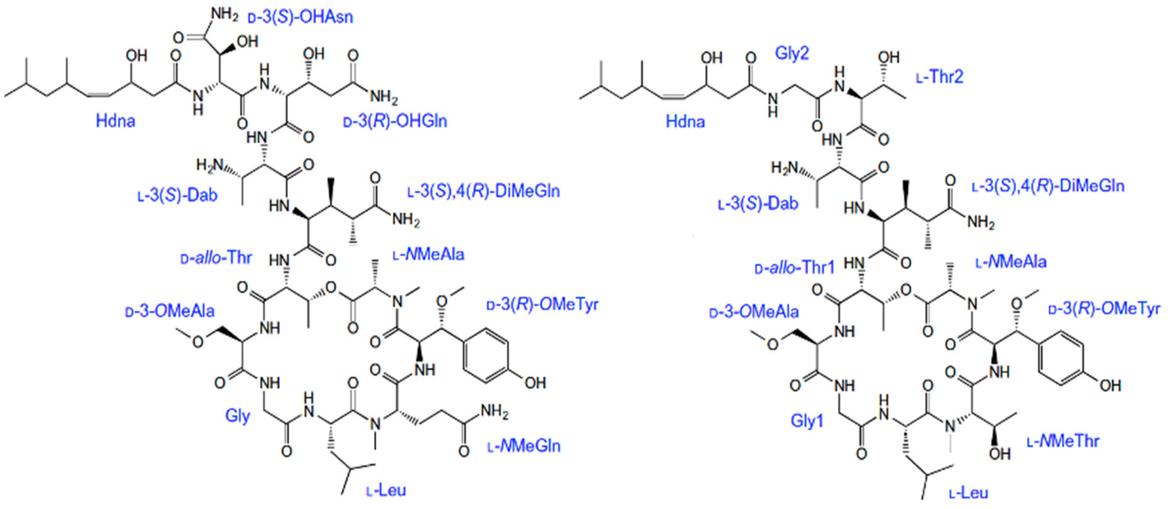

Figure S7. Stellattapeptins A (**134**) and B (**135**) isolated from *Stellatta* sp. found in North-western Australia [2].

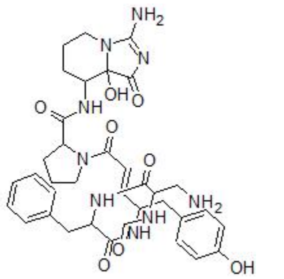

Pseudotheonamide C (**171**)
100964480

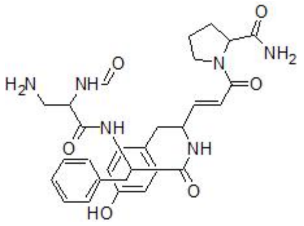

Pseudotheonamide D (**172**)
100964481

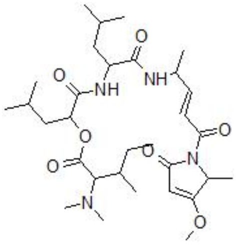

Gallinamide A (**173**)
25209862

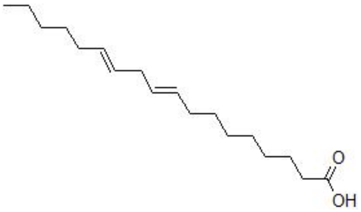

Linoleic acid (**179**)
5280450

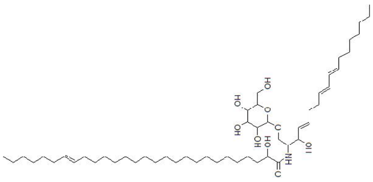

Renieroside A1 (**182**)
23643433

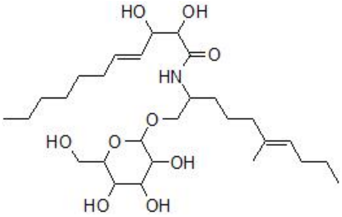

Penicilloside B (**184**)
156581282

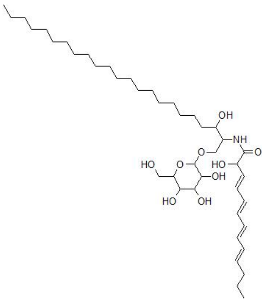

Asperiamide B (**185**)
139587498

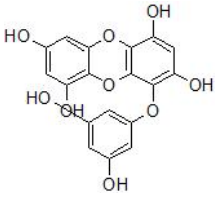

Eckol (**186**)
145937

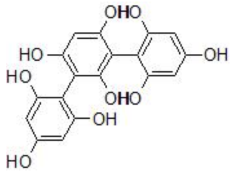

Trifucol (**188**)
71401226

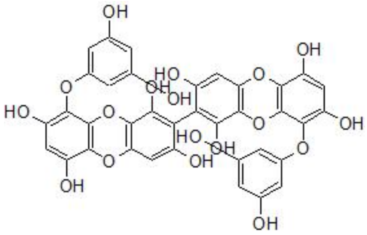

8,8-bieckol (**188**)
3008867

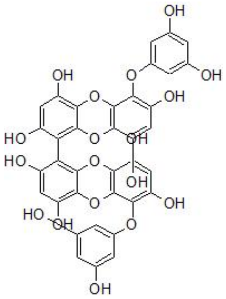

6,6-bieckol (**189**)
137388

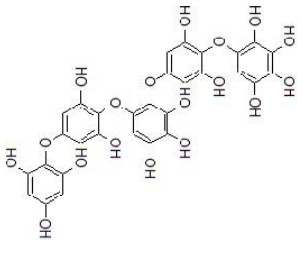

Hydroxypentafuhalol A (**194**) 102274236

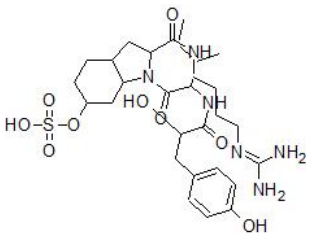

Aeruginosin 98B (**196**)
444346

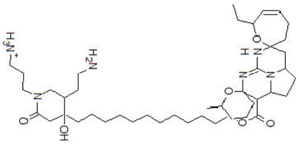

Crambescidin 786 (**204**)
132937446

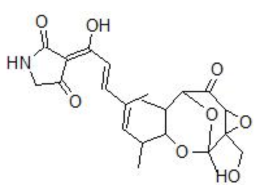

Isotirandamycin B (**206**)
145720669

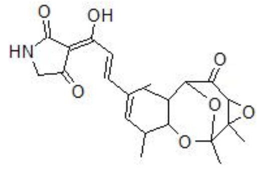

Tirandamycin A (**207**)
54706137

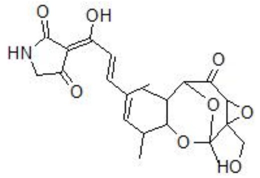

Tirandamycin B (**208**)
54728535

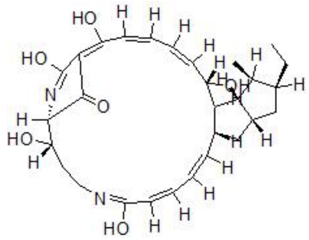

Alteramide A (**209**)
139584796

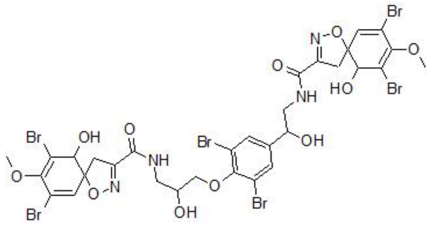

Isofistularin-3 (**210**)
159041

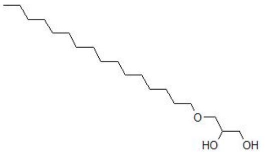

Chimyl alcohol (**211**)
72733

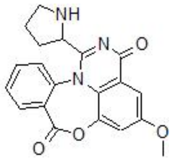

Aspergicin (**212**)
56949729

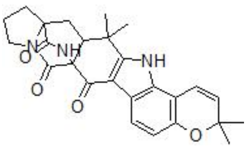

Notoamide I (**215**)
25180708

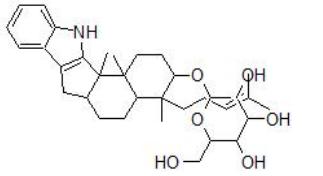

Emindole SB beta-mannoside (**216**)
76317959

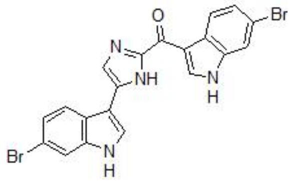

Dibromodeoxytopsentin (**218**) 11363725

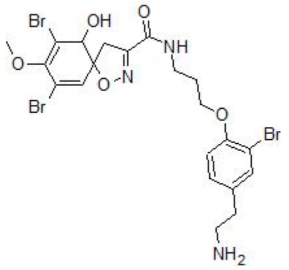

14-debromoaraplysillin I (**219**)
131984

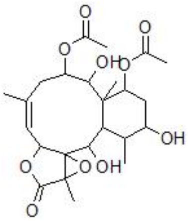

Excavatolide M (**226**)
10600828

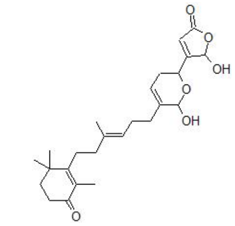

Fasciospongide A (**228**)

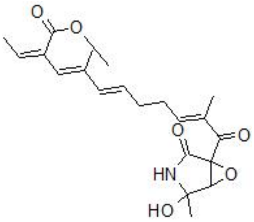

Epolactaene (**229**)
6442272

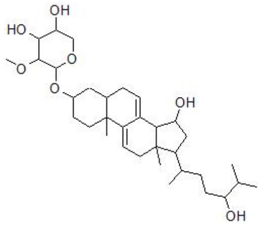

Moniloside A (**230**)
44575900

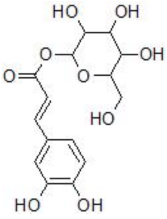

Caffeic acid hexoside (**231**)
6124135

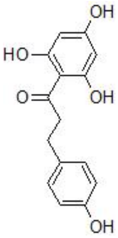

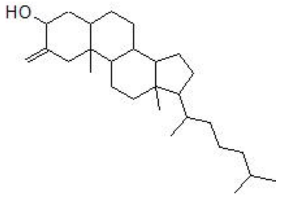

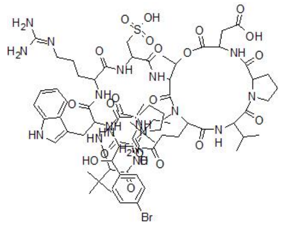

Phloretin (**232**)
4788

Cholestan-3-ol, 2-methylene-(3beta, 5 alpha) (CMBA) (**233**)
22213932

Microspinosamide (**240**)
25076067

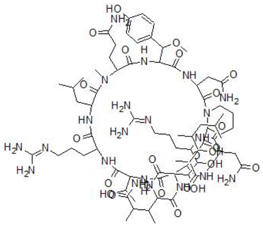

Neamphamide A (**241**)
25088478

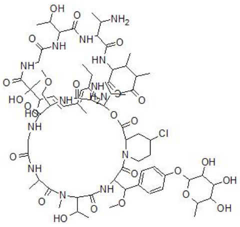

Mirabamide A (**242**)
24179735

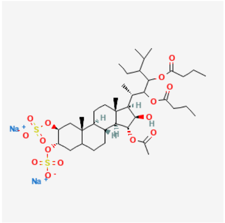

Clathsterol (**243**)
10887454

Figure S8. Chemical structures of identified compounds from marine organisms with inhibitory properties against SARS-CoV-2.

Table S1. Selected marine compounds with potential inhibitory properties against SARS-CoV-2 in silico.

Compound and structural formula. (PUBCHEMID)	Class of compound	Source	Target site	Binding Energy ΔG$_B$ (kcal/mo)	Refs.
Fistularin-3/11-epi-fistularin-3 (**245**) $C_{31}H_{30}Br_6N_4O_{11}$ (11170714) 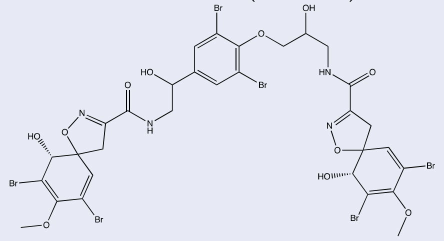	Alkaloid	Marine sponge Family Aplysinidae	Mpro	−7.8	[4]
15-α-methoxypuupehenol (**246**) $C_{22}H_{32}O_4$ (21591485) 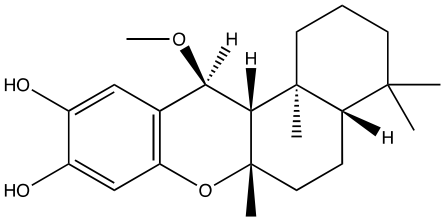	Phenol	Marine sponge *Petrosia strongylophora* sp.	Mpro	−7.2	[4]
Palmitoleic Acid acid (**247**) $C_{16}H_{30}O_2$ (445638) 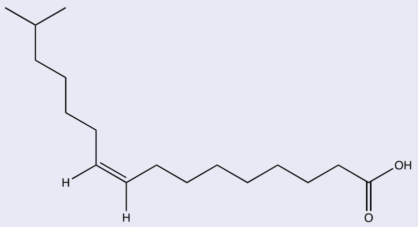	Lipid	Soft coral *Pterogorgia citrina*	Mpro	−7.5	[4]
(Hexadecyloxy) propane,1,2-diol (**248**) $C_{19}H_{40}O_3$ (21646261) 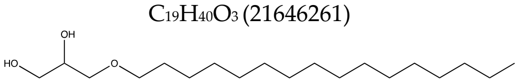	Lipid	Marine sponge Family Aplysinidae	Mpro	−7.5	[4]
Puupehedione (**249**) $C_{21}H_{26}O_3$ (460087) 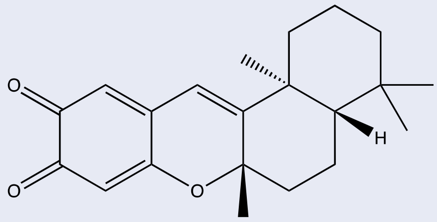	Terpene	Marine sponge *Petrosia strongylophora sp*	Mpro	−7.2	[4]
Glycosaminoglycan (**250**) $C_8H_{15}NO_9S$ (10053416)	Sulfonated polysaccharides	Marine Bacteria *Pseudomonas* sp.	Mpro	−7.9	[5]

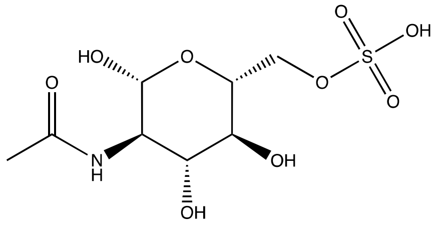					
Polyacetylenetriol (**251**)<br>$C_{29}H_{30}O_3$ (6474656)<br>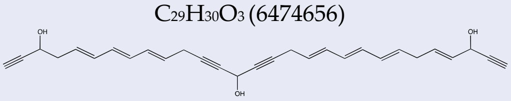		Marine Sponge *Petrosia* sp.,	Mpro	-7.4	[5,6]
Dehydrofurodendin (**252**)<br>$C_{22}H_{28}O_3$ (5273523)<br>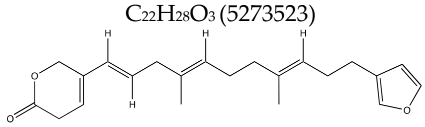	Furanoterpene	Marine Sponge *Lendenfeldia* sp.	Mpro	-6.2	[5]
Esculetin ethyl ester (**203)**<br>$C_{12}H_{10}O_6$ (11988319)<br>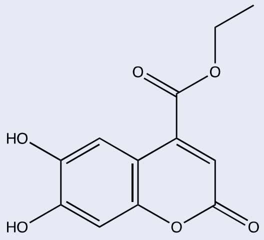	Polyketide	Marine Sponge *Axinella cf. corugata*	Mpro	-8.4	[5,7]
Dolabelladienetriol (**253**)<br>$C_{20}H_{34}O_3$ (6477027)<br>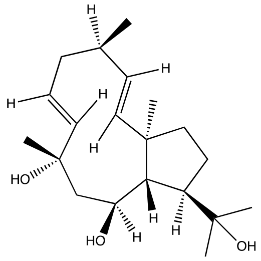	Terpenoid	Marine alga *Dictyota pfaffii*	Mpro	-7.6	[5]
Hamigeran b (**227)**<br>$C_{18}H_{21}BrO_3$ (9976066)<br>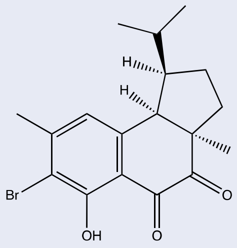	Diterpene	Marine sponge *Hamigera tarangaensis*	Mpro	-7.9	[5]
Phycocyanobilin (**174**)<br>$C_{33}H_{38}N_4O_6$ (5288007)	Phycobilin pigment	Cyanobacteria *Arthrospira* spp	Mpro<br>RdRp<br>PLpro	-8.6<br>-9.3<br>-9.8	[8–10]

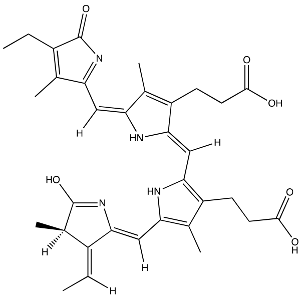					
Dictyosphaeric acid A (**225**)<br>$C_{22}H_{24}O_8$ (21585555)<br>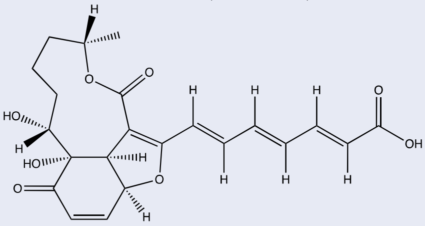	Polyketide decalactone	Green algae *Dicty-osphaeria ver-sluyii*	TMPRSS 2	−14.0	[11]
Excavatolide M (**226**)<br>$C_{24}H_{34}O_{10}$ (10600828)<br>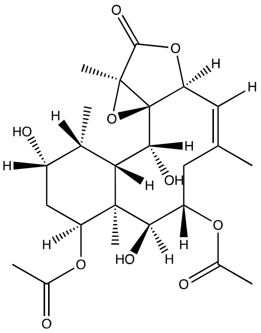	Diterpene	Soft coral *Briareum excavatum*	TMPRSS 2-S, PPIs, hACE2-S	−14.3	[11]
Apigenin-7-O-neohesperidoside (**200**)<br>$C_{27}H_{30}O_{14}$ (5282150)<br>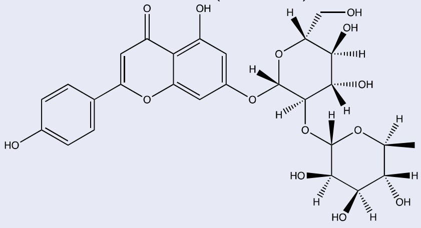	Flavonoid	Brown algae *Sargassum spinuligerum*	Mpro	−12.4	[12]

Luteolin-7-rutinoside (**201**) $C_{27}H_{30}O_{15}$ (10461109) 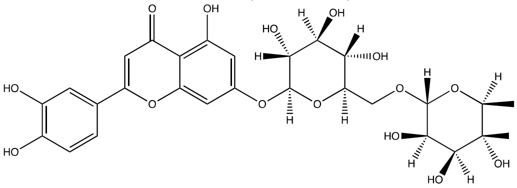	Flavonoid	Brown algae *Sargassum spinuligerum*	Mpro	12.1	[12]
Resinoside B (**197**) $C_{31}H_{34}O_{13}$ (10053416) 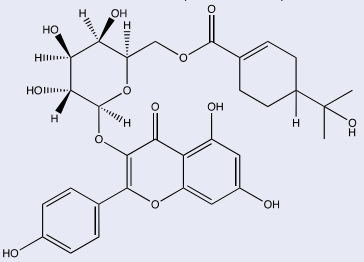	Flavonoid	Brown algae *Sargassum spinuligerum*	Mpro	−12.2	[12]
Didemnin A (**168**) $C_{49}H_{78}N_6O_{12}$ (123844) 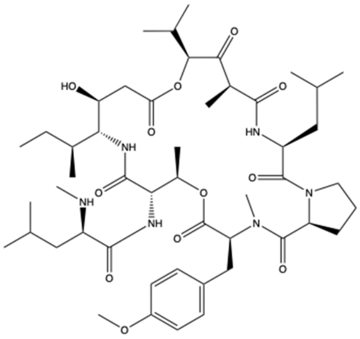				−11.8	
Didemnin B (**169**) $C_{57}H_{89}N_7O_{15}$ (122651) 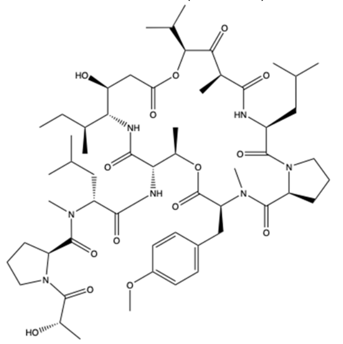	Depsipeptide	Tunicate *Trididemnum solidum*	Mpro	−10.2	[13]
Didemnin C (**170**) $C_{52}H_{82}N_6O_{14}$ (9963211)					

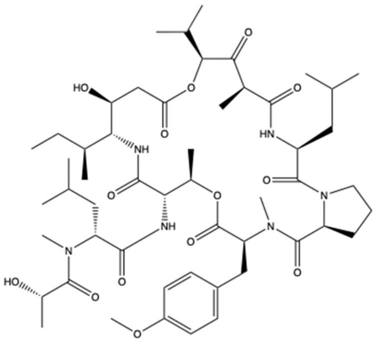				−9.2	
Heptafuhalol A (**190**)<br>(274129944)<br>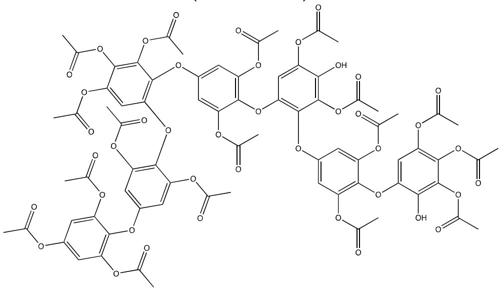				−14.6	
Phlorethopentafuhalol B (**192**)<br>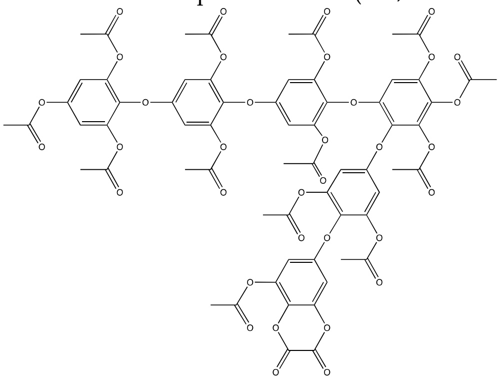	Phlorotannin	Brown algae<br>*Sargassum spinuligerum*	Mpro	−14,2	[12]
Pseudopentafuhalol C (**193**)<br>$C_{30}H_{22}O_{17}$ (101938042)<br>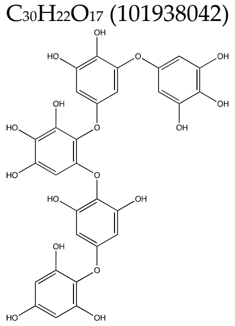				−14,2	

Eicosapentaenoic acid (**181**)<br>$C_{20}H_{30}O_2$ (446284)<br>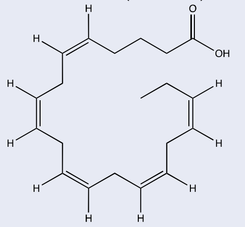	Lipids<br>(ω-6 PUFA)	Fish, other seafoods and algae	S-protein via binding to the FABP	−120.1	[14]
Docosahexaenoic acid (**180**)<br>$C_{22}H_{32}O_2$ (445580)<br>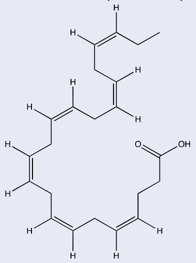				−110.8	

ID_{50} is ineffective dose, EC_{50} is Half maximal effective concentration, IC_{50} is Half maximal inhibitory concentration, FABP is fatty acid-binding pocket.

References

1. Niu, S.; Liu, D.; Shao, Z.; Proksch, P.; Lin, W. Eutypellazines A–M, Thiodiketopiperazine-Type Alkaloids from Deep Sea Derived Fungus Eutypella Sp. MCCC 3A00281. *RSC Adv* **2017**, *7*, 33580–33590, doi:10.1039/C7RA05774A.
2. Shin, H.J.; Rashid, M.A.; Cartner, L.K.; Bokesch, H.R.; Wilson, J.A.; McMahon, J.B.; Gustafson, K.R. Stellettapeptins A and B, HIV-Inhibitory Cyclic Depsipeptides from the Marine Sponge Stelletta Sp. *Tetrahedron Lett* **2015**, *56*, 4215–4219, doi:10.1016/J.TETLET.2015.05.058.
3. Wijanarko, A.; Lischer, K.; Hermansyah, H.; Pratami, D.K.; Sahlan, M. Antiviral Activity of Acanthaster Planci Phospholipase A2 against Human Immunodeficiency Virus. *Vet World* **2018**, *11*, 824, doi:10.14202/VETWORLD.2018.824-829.
4. Khan, M.T.; Ali, A.; Wang, Q.; Irfan, M.; Khan, A.; Zeb, M.T.; Zhang, Y.-J.; Chinnasamy, S.; Wei, D.-Q. Marine Natural Compounds as Potents Inhibitors against the Main Protease of SARS-CoV-2—a Molecular Dynamic Study. *J Biomol Struct Dyn* **2020**, *39*, 3627–3637, doi:10.1080/07391102.2020.1769733.
5. Vijayaraj, R.; Altaff, K.; Rosita, A.S.; Ramadevi, S.; Revathy, J. Bioactive Compounds from Marine Resources against Novel Corona Virus (2019-NCoV): In Silico Study for Corona Viral Drug. *Nat Prod Res* **2020**, *35*, 5525–5529, doi:10.1080/14786419.2020.1791115.
6. Fayed, M.A.A.; El-Behairy, M.F.; Abdallah, I.A.; Abdel-Bar, H.M.; Elimam, H.; Mostafa, A.; Moatasim, Y.; Abouzid, K.A.M.; Elshaier, Y.A.M.M. Structure- and Ligand-Based in Silico Studies towards the Repurposing of Marine Bioactive Compounds to Target SARS-CoV-2. *Arabian Journal of Chemistry* **2021**, *14*, 103092, doi:10.1016/j.arabjc.2021.103092.
7. De Lira, S.P.; Seleghim, M.H.R.; Williams, D.E.; Marion, F.; Hamill, P.; Jean, F.; Andersen, R.J.; Hajdu, E.; Berlinck, R.G.S. A SARS-Coronovirus 3CL Protease Inhibitor Isolated from the Marine Sponge Axinella Cf. Corrugata: Structure Elucidation and Synthesis. *J Braz Chem Soc* **2007**, *18*, 440–443, doi:10.1590/s0103-50532007000200030.

8. Pendyala, B.; Patras, A. In Silico Screening of Food Bioactive Compounds to Predict Potential Inhibitors of COVID-19 Main Protease (Mpro) and RNA-Dependent RNA Polymerase (RdRp). *ChemRxiv* **2020**, doi:10.26434/chemrxiv.12051927.v1.
9. Pendyala, B.; Patras, A.; Dash, C. Phycobilins as Potent Food Bioactive Broad-Spectrum Inhibitors Against Proteases of SARS-CoV-2 and Other Coronaviruses: A Preliminary Study. *Front Microbiol* **2021**, *12*, 1–10, doi:10.3389/fmicb.2021.645713.
10. Petit, L.; Vernès, L.; Cadoret, J.P. Docking and in Silico Toxicity Assessment of Arthrospira Compounds as Potential Antiviral Agents against SARS-CoV-2. *J Appl Phycol* **2021**, *33*, 1579–1602, doi:10.1007/s10811-021-02372-9.
11. Rahman, N.; Basharat, Z.; Yousuf, M.; Castaldo, G.; Rastrelli, L.; Khan, H. Virtual Screening of Natural Products against Type II Transmembrane Serine Protease (TMPRSS2), the Priming Agent of Coronavirus 2 (SARS-CoV-2). *Molecules* **2020**, *25*, 2271, doi:10.3390/molecules25102271.
12. Gentile, D.; Patamia, V.; Scala, A.; Sciortino, M.T.; Piperno, A.; Rescifina, A. Putative Inhibitors of SARS-COV-2 Main Protease from a Library of Marine Natural Products: A Virtual Screening and Molecular Modeling Study. *Mar Drugs* **2020**, *18*, doi:10.3390/md18040225.
13. Sahin, S.; Calapoğlu, F.; Ozmen, I. Didemnins Inhibit COVID-19 Main Protease (MPRO). *Biointerface Res Appl Chem* **2021**, *11*, 8204–8209, doi:10.33263/BRIAC111.82048209.
14. Hathaway, D.; Pandav, K.; Patel, M.; Riva-Moscoso, A.; Singh, B.M.; Patel, A.; Min, Z.C.; Singh-Makkar, S.; Sana, M.K.; Sanchez-Dopazo, R.; et al. Omega 3 Fatty Acids and COVID-19: A Comprehensive Review. *Infect Chemother* **2020**, *52*, 478–495, doi:10.3947/IC.2020.52.4.478.
